# Beyond basic taste: mechanisms, measurement, and food-matrix translation of non-basic oral sensations

**DOI:** 10.3389/fnut.2026.1962396

**Published:** 2026-10-23

**Authors:** Mehwish Ameer, Samina Saeed, Muhammad Shahbaz Zafar, Mingquan Huang

**Affiliations:** 1 School of Food and Health, Beijing Technology and Business University, Beijing, China; 2 Key Laboratory of Brewing Molecular Engineering of China Light Industry, Beijing Technology and Business University, Beijing, China; 3 Interdisciplinary Research Center for One Health, University of Sargodha, Sargodha, Pakistan

**Keywords:** astringency, chemesthesis, food-matrix translation, non-basic oral sensations, oleogustus, oral tribology, TRP channels

## Abstract

The five basic tastes–sweet, sour, salty, bitter and umami–account for only part of food acceptance and quality. Non-basic oral sensations, including astringency, pungency, cooling, metallic sensation, fatty mouthfeel, oleogustus, tingling, numbing and kokumi, contribute substantially to hedonic and textural perception and are commercially important, yet remain less well characterized. This integrative critical review synthesizes mechanistic, neurophysiological, analytical and applied evidence for these nine sensations, treating pungency as two mechanistically distinct pathways, TRPV1- and TRPA1-mediated, to give ten mechanistic entries. Each entry is assessed on two distinct axes: mechanistic confidence, how strongly the principal proximal mechanism is supported by available evidence, and food-matrix translation confidence, how reliably that mechanism predicts perception in real foods. The two axes are related but not equivalent: higher confidence in the principal proximal mechanism does not by itself ensure high confidence in food-matrix translation. TRPV1-mediated pungency, TRPM8-mediated cooling, astringency and fatty mouthfeel rest on well-supported principal or proximal mechanisms, yet none of the ten entries reaches high translation confidence, while TRPA1-mediated pungency, tingling, numbing, oleogustus and kokumi are weaker on both axes. Three distinctions are emphasized: pungency is not a single mechanism; fatty mouthfeel is lubrication-based and mechanoreceptor-mediated, whereas oleogustus is a proposed fatty-acid-receptor perception that remains debated; and iron-related metallic sensation arises largely from retronasal perception of lipid-oxidation volatiles, with no specific human metallic receptor identified. The principal conclusion is that a well-supported mechanism does not by itself confer predictive power in food: matrix composition, salivary dilution and oral processing reshape perception, so dynamic and tribological methods are more ecologically valid than static intensity ratings, and these sensations warrant greater consideration in product formulation and sensory evaluation.

## Highlights

- Non-basic oral sensations are mechanistically heterogeneous and require integrated frameworks spanning food chemistry, oral tribology, and trigeminal neuroscience.
- Fatty mouthfeel (tribological) and oleogustus (proposed FFA-receptor-mediated) are distinct; CD36/GPR120 relate to oleogustus, not the lubrication-based component of fatty mouthfeel.
- Iron-induced metallic off-flavor is best explained by retronasal lipid-oxidation volatiles; no specific human metallic taste receptor has been identified.
- Evidence is graded on two distinct axes: well-supported principal mechanisms (TRPV1, TRPM8, astringency, fatty mouthfeel) still translate only moderately to real food, and no entry reaches high confidence on both axes.
- Static ratings undercharacterize dynamic sensations; oral tribology, time-intensity, and temporal dominance of sensations are needed for astringency, pungency, and cooling.

## 1 Introduction

Consumer acceptance of food is influenced by the integrated perception of multiple, concurrent sensory signals. Although regulatory frameworks and much of taste science have historically focused on canonical basic tastes, many sensory attributes that determine food quality and consumer acceptance fall outside this traditional framework. Beyond the five classic taste sensations, astringency from red wine, capsaicin’s burning sensation, the cooling sensation of menthol, the silky lubrication of well-emulsified fat, and the diffuse mouthfulness of fermented foods can play an important role in product design and consumer preference (1, 2). These perceptions are a result of the multisensory integration of the olfactory, gustatory, trigeminal and somatosensory inputs, which interact at both the perceptual and neural levels (3, 4).

Non-basic oral sensations are relevant to food formulation. Astringency, dryness, and off-flavors are common characteristics of plant-based protein foods which can negatively affect sensory acceptance (5, 6). Fat reduction is often problematic because it alters lubrication, rheological properties, and sensory perception (7, 8). Perceived pungency in spiced foods depends on partitioning within the matrix, on the release of sensory-active compounds, and on interactions between activating and suppressing constituents (9–11). The retronasal lipid-oxidation products and other incompletely resolved oral pathways generated by iron fortification can create metallic off-flavors (12, 13).

In food systems, perception of non-basic oral sensations is further shaped by formulation-related variables that influence stimulus delivery and sensory integration (7–9, 14–17). Factors such as texture, particle size, viscosity, emulsion structure, fat distribution, and interactions between flavor compounds and food components can alter the release, transport, and perception of sensory-active molecules (7, 8, 14, 17). For example, the same concentration of a pungent compound, polyphenol, or lipid-derived stimulus may produce different sensory responses depending on matrix composition, rheological properties, and oral processing behavior (9, 11–13). Therefore, understanding non-basic oral sensations requires integration of molecular mechanisms with food structure, sensory measurement, and human perception rather than evaluation of isolated compounds alone.

Non-basic oral sensations, however, appear to be comparatively fragmented in the literature in spite of their growing importance. First, they are often confused with taste, which can obscure their distinct neural bases, sensory mechanisms, and temporal characteristics. For instance, astringency is currently best understood as a tactile–tribological sensation and is more likely due to polyphenol–salivary-protein interactions and oral friction changes than taste receptor cell activation, although the final transduction mechanisms in the nerves involved remain unclear. Metallic sensations are mediated by an integration of gustatory, olfactory and somatosensory inputs, while “cooling” is mediated primarily by thermosensitive pathways and not by classical taste mechanisms. Second, the understanding of the mechanisms is still highly dispersed in the fields of food chemistry, oral biology, sensory psychophysics and trigeminal neuroscience. Third, many of these sensations vary over time, requiring analytical techniques that capture onset, peak, persistence, and carry-over; static intensity ratings cannot adequately characterize them (16), and *in vitro* assays do not necessarily mimic the salivary, thermal, mechanical, and frictional conditions of the oral environment.

Despite substantial advances in identifying molecular and sensory mechanisms for individual non-basic oral sensations, the current evidence base remains fragmented across food chemistry, sensory science, oral physiology, and neuroscience. Significant progress has been achieved in identifying receptor-level mechanisms for chemesthetic sensations, physicochemical interactions underlying astringency, and candidate pathways involved in flavor modulation; however, these advances have developed largely within individual disciplinary frameworks. A major unresolved challenge is that mechanistic identification does not necessarily translate into reliable prediction of perception in real food matrices. Receptor activation, molecular interactions, and physicochemical measurements obtained under simplified conditions may not fully account for the effects of salivary composition, matrix partitioning, oral processing, temporal dynamics, and sensory integration that determine perception during food consumption. Therefore, a central challenge in this field is to establish how mechanistic knowledge can be translated into predictive understanding of oral sensations in complex food systems. This review provides a critical and comparative analysis of nine target non-basic oral sensations: astringency, pungency, cooling, metallic sensation, fatty mouthfeel, oleogustus, tingling, numbing, and kokumi.

A key contribution of this review is the introduction of two distinct evidence dimensions, mechanistic confidence and food-matrix translation confidence, which are related but not interchangeable. These nine sensations are grouped into seven practical organizing categories in section “3 Classification of non-basic oral sensations.” Consequently, Tables 1, 2 contain ten mechanistic entries representing nine target sensations, with pungency separated into TRPV1- and TRPA1-mediated pathways because these mechanisms represent distinct chemesthetic processes. The review assesses the quality and consistency of evidence available on proposed mechanisms, highlights unresolved questions and draws a line between existing mechanistic understanding and practical predictive ability for real foods. Rather than simply summarizing reported findings, this review compares competing mechanistic explanations, evaluates limitations of current approaches, and identifies priorities for future research. Specifically, this review aims to (i) classify non-basic oral sensations according to their dominant sensory and molecular mechanisms; (ii) evaluate the strength of evidence linking proposed mechanisms with human perception; (iii) identify limitations of current analytical and sensory approaches; and (iv) highlight future directions for improving translation between mechanistic discoveries and food-matrix applications. These two confidence axes are applied throughout the manuscript to evaluate how strongly mechanisms are supported by the available evidence and how reliably they predict perception in real food matrices. Mechanistic confidence is classified as HIGH, MODERATE, or DEBATED, reflecting how strongly the principal receptor, physical pathway, or other proximal mechanism is supported by the available evidence. Food-matrix translation confidence is classified as HIGH, MODERATE, or LOW, reflecting how reliably the proposed mechanism predicts human sensory perception in real food matrices.

**TABLE 1:** Classification of non-basic oral sensations: perceptual descriptors, principal stimuli, main receptor/pathway, food examples, and key measurement methods.

Sensation	Perceptual descriptors	Principal stimuli	Main receptor/pathway	Food examples	Key measurement methods
Astringency	Drying, puckering, roughness, grittiness	Condensed tannins, catechins, EGCG, proanthocyanidins	Polyphenol–salivary protein interactions → reduced lubrication; neural transduction remains unresolved (26, 27, 34, 37, 43).	Red wine, tea, cocoa, legumes, berries	Oral tribology (Stribeck curve), SDS-PAGE, TI, TDS
Pungency (TRPV1)	Burning, stinging, heat, irritation	Capsaicin, gingerols, piperine, shogaols	TRPV1 activation on trigeminal C-fibers; dual gating by capsaicin and noxious heat >43 °C (19, 24).	Chili, ginger, black pepper	TI, psychophysics, TRPV1 cell assay, TDS
Pungency (TRPA1)	Sharpness, nasal irritation, bite	Allyl isothiocyanate, cinnamaldehyde, allicin	TRPA1 activation by electrophilic agonists via reactive cysteine modification; non-electrophilic activation differs (21, 22).	Mustard, wasabi, cinnamon, garlic	Threshold studies, TI, TRPA1 cell assay, GC-MS
Cooling	Cool, fresh, minty, mild numbness	Menthol, eucalyptol, WS-3, WS-23, Coolact P	TRPM8 activation by menthol and other cooling agents (20, 24).	Mint products, chewing gum, cooling beverages	Threshold, TI, QDA, psychophysics
Metallic sensation	Blood-like, rusty, mineral	Fe^2+^/Fe^3+^ (primary); lipid-oxidation volatiles (1-octen-3-one, 1-nonen-3-one)	Metal-catalyzed lipid oxidation and retronasal olfaction; possible trigeminal/gustatory contributions; no specific human metallic receptor identified (12, 13, 55).	Fortified beverages, tap water, oxidized fats	Descriptive analysis, GC-O, GC-MS, nasal-occlusion comparison
Fatty mouthfeel	Creamy, oily, coating, smooth, rich	Triglycerides, emulsified fat droplets	Tongue–palate lubrication and oral mechanosensation; distinct from oleogustus (7, 8, 14, 17).	Dairy products, emulsions, butter, cream	Oral tribology, rheology, QDA, TDS
Oleogustus	Rancid-like, fatty, thick	Long-chain FFAs (oleic, linoleic, palmitic acids)	Proposed CD36/GPR120-mediated fatty-acid sensing (50, 51); classification as taste vs. satiety signal remains debated.	Aged/rancid oils, blue cheese, dark chocolate	Threshold detection, forced-choice discrimination, nasal-occlusion control, GC-MS
Tingling	Fizzy, electric, buzzing, paresthesia	Hydroxy-α-sanshool (HAS)	KCNK3/KCNK9/KCNK18 inhibition contributes to mechanoreceptor depolarisation; TRPA1/TRPV1 contribution remains uncertain (30, 31).	Szechuan pepper	Quantitative sensory testing, TI, TDS
Numbing	Anesthetic, deadening, loss of tactile sensation	Hydroxy-α-sanshool (high concentration)	Associated with KCNK modulation; numbing-specific transduction remains unresolved (30, 31, 56).	Szechuan pepper (málà cuisine)	Quantitative sensory testing, TDS
Kokumi	Mouthfulness, thickness, richness, continuity	γ-Glu-Val-Gly, glutathione, γ-glutamyl peptides	Proposed CaSR-mediated modulation of taste signaling; exclusivity and predictive relevance remain unresolved (32, 57, 61).	Fish sauce, soy sauce, aged cheese, yeast extract	Trained-panel difference tests, CATA, CaSR cell assay

Representative studies supporting the receptor/pathway assignments are cited directly in the main receptor/pathway column. TI, time-intensity; TDS, temporal dominance of sensations; QDA, quantitative descriptive analysis; GC-MS, gas chromatography–mass spectrometry; GC-O, gas chromatography–olfactometry; PRP, proline-rich protein; FFA, free fatty acid; CaSR, calcium-sensing receptor. Fatty mouthfeel and oleogustus are mechanistically distinct: the lubrication-based component of fatty mouthfeel does not require a taste-receptor mechanism (which does not exclude interactions with fatty-acid sensing), whereas oleogustus is a proposed FFA-receptor-mediated perception that remains debated (94). CD36 and GPR120 are listed under oleogustus only. The review considers nine target oral sensations. Pungency is represented by two mechanistically distinct entries–TRPV1-mediated and TRPA1-mediated pungency–resulting in ten rows in this table.

**TABLE 2:** Two-axis evidence-confidence assessment of ten mechanistic entries representing nine non-basic oral sensations.

Sensation	Principal compound(s)	Transduction pathway	Mechanistic confidence	Translation confidence	One-sentence justification/Key limitations
Astringency	Condensed tannins, catechins, EGCG	Polyphenol–PRP/mucin interactions → lubrication loss → mechanoreceptor activation (specific receptor unidentified); TRPV2/TRPC5 remain candidates supported by heterologous evidence.	HIGH	MODERATE	The proximal polyphenol–salivary-protein/lubrication mechanism is well supported, but downstream neural transduction and translation across complex food matrices remain incomplete (6, 26, 27, 37, 43, 83).
Pungency (TRPV1)	Capsaicin, gingerols, piperine	TRPV1 activation on trigeminal C-fibers; reversible agonist binding	HIGH	MODERATE	TRPV1-mediated capsaicin sensing is well established, but matrix composition and endogenous suppressors modify food pungency, and receptor-assay prediction remains limited to narrow product sets (9, 11, 19, 48).
Pungency (TRPA1)	Allyl isothiocyanate, cinnamaldehyde, allicin	Covalent modification of reactive cytoplasmic cysteines by electrophilic agonists	MODERATE	LOW	Electrophilic activation through reactive cysteine modification is supported, but other activation routes and human food-matrix translation remain incompletely characterized (21, 22, 47).
Cooling	Menthol, WS-3, WS-23, Coolact P	TRPM8 activation; menthol sensitizes the channel to cold, enabling activation at warmer temperatures	HIGH	MODERATE	TRPM8-mediated cooling is well established, but biosensor–sensory correspondence derives mainly from simplified systems, limiting prediction in finished foods (20, 49, 81).
Metallic sensation	Fe^2+^ (primary); Cu^2+^, Zn^2+^ (secondary)	Metal-catalyzed lipid oxidation → retronasal volatiles (dominant for Fe); possible minor trigeminal/gustatory contributions; no specific taste receptor identified	MODERATE	MODERATE	Human nasal-occlusion and volatile studies support a major retronasal pathway for iron, but perception varies across metals and no dedicated human metallic receptor has been established (12, 13, 55).
Fatty mouthfeel	Triglycerides, emulsified fat	Elastohydrodynamic lubrication → oral mechanoreceptor activation (population unidentified); does not require taste receptors or exclude fatty-acid sensing.	HIGH	MODERATE	Tribological and sensory evidence supports lubrication as a major contributor, but the relevant mechanoreceptors and prediction of the full multidimensional fat percept remain unresolved (7, 8, 14, 17).
Oleogustus	Long-chain FFAs (oleic, linoleic, palmitic acids)	Proposed: CD36 and GPR120/FFAR4 on taste-bud cells; taste vs. satiety role unresolved; GPR40 largely animal evidence	DEBATED	LOW	Human psychophysical and taste-cell evidence supports FFA sensing involving CD36/GPR120, but receptor sufficiency, olfactory confounding, and basic-taste status remain unresolved (29, 50, 51, 53, 54).
Tingling	Hydroxy-α-sanshool (HAS)	KCNK3/KCNK9/KCNK18 inhibition → mechanoreceptor depolarisation; any TRP contribution at high concentrations is minor	MODERATE	LOW	KCNK inhibition is supported by mechanistic and human psychophysical evidence, but translation to whole-food matrices remains poorly characterised (30, 31).
Numbing	HAS (high concentration), alkylamides; coumarins (e.g., pomelo peel)	Less securely resolved than tingling; associated with KCNK modulation, no established numbing-specific pathway	DEBATED	LOW	Sanshool-related KCNK modulation is implicated, but no numbing-specific pathway is established and chemically distinct food compounds can produce similar effects (30, 31, 56).
Kokumi	γ-Glu-Val-Gly, glutathione, γ-glutamyl peptides	Proposed: CaSR activation in type II taste-bud cells → paracrine modulation; may also act through broader taste interactions	MODERATE	LOW	CaSR involvement is supported by receptor, structural and human antagonist evidence in model solutions, and γ-Glu-Val-Gly has demonstrated sensory effects in food; however, CaSR exclusivity and sufficiency in human kokumi perception and broad food-matrix prediction remain unresolved (32, 57, 59, 61–63).

Pungency is separated into TRPV1- and TRPA1-mediated pathways. Ratings follow the predefined criteria in Table 3; concise supporting evidence and key limitations are shown here, with the full evidence base provided in Supplementary Table 1. Mechanistic and food-matrix translation confidence follow the predefined qualitative decision criteria in Table 3 and represent author-assigned classifications. PRP, proline-rich protein; FFA, free fatty acid; CaSR, calcium-sensing receptor; KCNK, two-pore-domain potassium channel. The terms “established,” “supported,” and “proposed” are narrative descriptors and do not constitute additional evidence categories.

### 1.1 Positioning of the present review relative to existing reviews

Several recent reviews address parts of this territory, including a broad review of mouthfeel physiology and biochemistry (14); a review of astringency in plant proteins (5); a tribo-chemical account of plant-based astringency (15); a review of astringency sub-qualities and saliva-lubrication measurement (16); a review of the oral lubrication performance of plant-derived polymers (17); and a systematic review linking oral tribology to sensory perception (8). Each provides depth within a particular sensation or mechanism. The contribution of the present review lies not in broader coverage alone, but in a cross-sensation, two-axis evaluation that distinguishes mechanistic confidence from food-matrix translation confidence and applies the same evidence-grading framework logic to every sensation category, making explicit where a well-supported proximal mechanism nevertheless fails to predict perception in food.

## 2 Review scope and literature selection

This is an integrative critical review, not a systematic review in the PRISMA sense. An integrative-review approach is appropriate for synthesizing evidence across heterogeneous study designs and disciplines (18). As this review was not designed as a systematic review, an exhaustive database screening log and formal inclusion count were not undertaken. Recent authoritative reviews and systematic reviews were used to synthesize broad evidence across disciplines, whereas foundational, controversial, quantitative, and mechanism-defining claims were checked against selected primary studies. The review also incorporates a structured update of recent primary and review literature published between 2020 and 2026, with sources selected according to their specific scientific role rather than through an exhaustive systematic search.

### 2.1 Databases and search strategy

Literature was identified primarily through Google Scholar, supplemented by institutional full-text access and direct retrieval from publisher websites, together with backward reference-list searching (snowballing) of key papers. Search terms covered each of the nine sensation categories, their principal mechanistic pathways (including TRP-channel pharmacology, oral tribology, polyphenol chemistry, lipid oxidation, CaSR signaling, and KCNK-channel inhibition) and relevant measurement approaches such as time-intensity, temporal dominance of sensations, CATA, tribology, GC-MS, and LC-HRMS. Emphasis was placed on published literature from 2015 onward, with older foundational studies retained selectively. No formal language restriction was applied as a filter, although the included literature is predominantly English-language. The search was targeted and iterative rather than exhaustive, and was refined as key papers and their reference lists were examined. The exact search strings and the final search date were not formally recorded and therefore cannot be reported reliably; they were not reconstructed retrospectively. Backward reference-list searching (snowballing) was the main route used to identify additional primary sources; forward citation searching was not systematically documented. No PRISMA screening flow, database screening log, or count of records was maintained, and such counts are not reported. To improve reproducibility, the search space is specified explicitly here rather than reconstructed as a retrospective log. Searches combined a sensation block (astringency OR pungency OR cooling OR “metallic taste” OR “fatty mouthfeel” OR oleogustus OR tingling OR numbing OR kokumi), a mechanism block (TRPV1 OR TRPA1 OR TRPM8 OR KCNK OR “two-pore potassium” OR CD36 OR GPR120 OR FFAR4 OR “calcium-sensing receptor” OR CaSR OR “salivary protein” OR “proline-rich protein” OR “lipid oxidation” OR tribology OR lubrication), and, where relevant, a measurement or matrix block (“time-intensity” OR “temporal dominance” OR CATA OR “descriptive analysis” OR psychophysics OR GC-MS OR LC-HRMS OR “food matrix” OR “plant-based”), with terms combined using OR within blocks and AND across blocks. These strings express the concept blocks applied during the iterative search rather than a verbatim record of each query executed. Because well-defined search strategies are recognized as central to rigor in integrative reviews (18), this limitation is stated explicitly: the search was purposive rather than exhaustive, and the evidence-confidence ratings reported in Table 2 therefore describe the strength of the evidence identified by this search rather than a guarantee that no further relevant study exists.

### 2.2 Inclusion and exclusion criteria

Papers were included if they reported original mechanistic, sensory, or applied data directly relevant to one or more of the sensation categories, were published in peer-reviewed journals, or represented foundational primary discoveries. Older studies were retained selectively, including foundational primary discoveries such as TRPV1 identification (19), TRPM8 identification (20), TRPA1 characterization (21) with its covalent-modification mechanism established separately (22), an early foundational kokumi study (23), and the retronasal metallic-taste study (12). Papers were excluded if only tangentially relevant or superseded by a more comprehensive recent treatment. Review papers were used principally for background framing; mechanistic, quantitative, and causal claims are supported by primary papers where those were available. High-risk, corrected, or recently updated reference entries were verified against publisher pages and DOI records. Study selection was purposive rather than protocol-driven: candidate sources were screened for direct relevance to a sensation category and for the evidence type required by the specific claim, with primary studies prioritized for mechanistic, quantitative, receptor-defining, and causal claims, and reviews and systematic reviews used mainly for broad synthesis (18). Duplicate records were identified and removed by title, authors, and DOI.

### 2.3 Evidence assessment: two distinct axes

A central argument of this review is that the evidence for a non-basic oral sensation cannot be captured on a single quality scale, because two distinct questions are frequently conflated. The first is mechanistic confidence: how strongly the principal receptor, physical pathway, or other proximal mechanism is supported by the available evidence. The second is food-matrix translation confidence: how reliably that mechanism predicts perceived intensity and character in real food, where salivary dilution, matrix partitioning, temperature, and oral processing intervene. A sensation may rest on a securely identified receptor yet translate poorly to food, while a sensation with only a partially resolved mechanism may nonetheless be managed reliably through empirical formulation. Across the ten entries assessed here, the two dimensions address related but distinct aspects of confidence: higher mechanistic confidence does not by itself ensure high confidence in food-matrix translation.

Throughout the manuscript, and in Tables 2, 3, each sensation is assessed on both axes using predefined qualitative decision criteria. Mechanistic confidence is classified as HIGH when the principal receptor, physical pathway, or other proximal mechanism is supported by convergent experimental evidence and has been reproducibly characterized; MODERATE when a candidate pathway is supported by experimental evidence but remains incomplete, context-dependent, or insufficiently resolved; and DEBATED when competing mechanisms remain plausible, evidence is inconsistent, or no consensus pathway has been established. A HIGH mechanistic-confidence rating refers to the strength of evidence supporting the principal proximal mechanism or principal pathway and does not imply complete resolution of downstream neural transduction or of the integrated sensory percept. Food-matrix translation confidence is classified as HIGH when consistent direct human sensory evidence links the proposed mechanism to perception in at least two distinct real-food contexts; MODERATE when direct human sensory evidence supports the mechanism–perception relationship but is confined to a single context, a narrow product range, or food-relevant model systems, so that generalization remains limited; and LOW when the mechanism–perception relationship itself remains unestablished in humans, when the available evidence derives predominantly from isolated compounds, model systems, or non-human studies, or when the evidence bears on the sensation without establishing the mechanism responsible for it. For this axis, demonstration of a sensory effect in a food matrix was not considered sufficient by itself; the evidence also had to inform the proposed relationship between the mechanism and the perceived sensation.

**TABLE 3:** Predefined decision criteria for the two-axis evidence-confidence framework.

Evidence axis	Confidence level	Predefined decision criterion
Mechanistic confidence	HIGH	The principal receptor, physical pathway, or other proximal mechanism is supported by convergent experimental evidence and has been reproducibly characterized.
	MODERATE	A candidate receptor or physical pathway is supported by experimental evidence but remains incomplete, context-dependent, or insufficiently resolved.
	DEBATED	Competing mechanisms remain plausible, evidence is inconsistent, or no consensus pathway has been established.
Food-matrix translation confidence	HIGH	Consistent direct human sensory evidence links the proposed mechanism to perception in at least two distinct real-food contexts.
	MODERATE	Direct human sensory evidence supports the mechanism–perception relationship but is confined to a single context, a narrow product range, or food-relevant model systems; generalization remains limited.
	LOW	The mechanism–perception relationship remains unestablished in humans; evidence derives predominantly from isolated compounds, model systems, or non-human studies, or bears on the sensation without establishing the mechanism responsible for it.

These criteria are qualitative decision rules developed for this review and are not intended to constitute a validated numerical evidence-scoring instrument.

Evidence was weighted according to its directness to the question being assessed. For mechanistic confidence, receptor-level, molecular, functional, causal, and replicated evidence was prioritized over indirect or descriptive evidence. For translation confidence, human psychophysical or food-sensory evidence obtained in real foods was prioritized over cell-based, animal, *ex-vivo*, *in vitro*, or computational evidence, and evidence spanning multiple food matrices was considered stronger than evidence from a single product. Each rating was assigned by applying these predefined criteria to the evidence identified for the relevant sensation, and the principal supporting evidence or limiting factor is reported in Table 2.

This framework is therefore a predefined qualitative decision framework rather than a validated quantitative scoring system. It is intended to make the basis of each classification explicit while preserving the distinction between mechanistic certainty and food-matrix translation.

The evidence ratings were assigned through author-level synthesis of the published evidence rather than by transferring confidence labels assigned by individual studies. For each sensation, the relevant mechanistic and human sensory/food-matrix findings reported in the included literature were examined against the predefined criteria in Table 3. The evidence supporting each rating was then documented in Table 2 through the corresponding justification and key limitations. Ratings were assigned as follows: the first author applied the predefined criteria in Table 3 to the evidence identified for each sensation and proposed an initial rating on each axis together with the supporting justification recorded in Table 2; the proposed ratings and justifications were then reviewed by the co-authors against the same criteria, and any disagreement was resolved by discussion with reference to the criteria and to the underlying studies until consensus was reached, so that the final ratings reported here are those agreed by all authors. The evidence underlying each rating is set out in full in Supplementary Table 1, which lists, for every entry, the rating on each axis, the key human sensory evidence, the key mechanistic or model-system evidence, the principal limitations bearing on the rating, and representative references, with evidence types labeled explicitly. Ratings were not assigned independently in duplicate by two or more assessors, and no inter-rater agreement statistic was therefore calculated; this is acknowledged as a limitation of the framework.

Priority was given to evidence directly addressing the relevant mechanistic pathway and, for translation confidence, to human sensory evidence obtained in real food matrices; indirect evidence from simplified or model systems was considered according to the limitations specified in the predefined criteria.

For reproducibility, the evidentiary basis and principal limiting considerations underlying each rating are reported in Table 2, with representative mechanistic, human sensory, and food-matrix studies cited directly for each classification; the decision rules used to assign the confidence categories are specified in Table 3.

## 3 Classification of non-basic oral sensations

For conceptual organization, the nine target oral sensations considered in this review are grouped into seven practical higher-order categories according to their predominant sensory and neurophysiological characteristics. These organizing categories are not equivalent to individual sensations or mechanistic pathways: most notably, pungency is represented by separate TRPV1- and TRPA1-mediated pathways (19, 21, 22), resulting in ten mechanistic entries across Tables 1, 2. Many oral sensations are multimodal and therefore resist completely discrete classification. Two limitations should be stated at the outset. First, several receptors are polymodal: TRPV1, for instance, transduces both a chemical stimulus (capsaicin) and a thermal one (noxious heat), so a strict substrate criterion cannot cleanly assign it to one category. Second, the criterion used to separate flavor modulators such as kokumi from taste qualities (that they produce no distinct percept in isolation) is operationally fragile, since some accepted basic tastes are also weak near threshold. These categories are therefore a practical organizing framework rather than mutually exclusive natural kinds.

### 3.1 Chemesthetic sensations

Chemesthesis refers to chemical stimulation of somatosensory receptors in the oral mucosa, producing sensations of pain, irritation, temperature, or pressure without tissue damage (24, 25). The primary mediators are TRP channels on trigeminal afferent fibers. The key distinction from gustation is that chemesthetic sensations are transduced not by taste-bud receptor cells but by afferent fibers also carrying nociceptive and thermoceptive signals (24, 25). Chemesthesis is defined here by the afferent pathway rather than by a single receptor family: TRP channels mediate the pungent and thermal categories treated in sections “3.1 Chemesthetic sensations” and “3.3 Chemically induced thermal sensations,” whereas the mechanosensory paresthesias discussed later in section “3.6 Mechanosensory paresthesia” involve distinct ion-channel mechanisms rather than classical TRP-mediated chemesthesis.

### 3.2 Tactile and friction-related sensations

This category encompasses sensations arising from changes in the physical interaction between food and oral surfaces, mediated by mechanoreceptors and low-threshold tactile afferents. Astringency is the defining member, arising from loss of salivary lubrication following polyphenol-induced precipitation or conformational alteration of salivary proteins (26, 27). The organizing framework for this category is oral tribology rather than taste-receptor chemistry (15, 28). A tribo-chemical perspective extends this by recognizing that chemical modification of salivary proteins and physical changes at the oral interface are simultaneous rather than sequential; neither dimension alone provides a complete account (15).

### 3.3 Chemically induced thermal sensations

TRPM8 mediates cooling sensations elicited by menthol and other cooling agents. Menthol sensitizes the channel to cold, allowing activation at warmer temperatures (20). TRPV1 mediates chemesthetic heat from capsaicin through the same activation gate used by noxious heat (19). These are chemesthetic rather than gustatory perceptions, carried by trigeminal afferents rather than by taste-receptor cells (25).

### 3.4 Metallic and mineral-like sensations

Metallic sensation is among the most poorly classified non-basic oral sensations. Current evidence supports a multimodal integrated-percept model: for iron, the best-evidenced pathway is metal-catalyzed lipid oxidation generating retronasal volatiles, with possible secondary contributions from trigeminal chemesthesis and gustatory epithelial interaction. Metallic perception is heterogeneous across metal species, with a particularly strong retronasal contribution documented for iron (12).

### 3.5 Fat-related oral sensations

Two mechanistically distinct fat-related sensations must be separated. Fatty mouthfeel arises from the physical properties of fat in the mouth (tribological lubrication, rheological viscosity, mucosal film formation) transduced by mechanoreceptors. The lubrication-based component of fatty mouthfeel does not require a taste-receptor mechanism; this does not exclude interactions with fatty-acid sensing or other sensory pathways. Oleogustus denotes the proposed taste-like quality elicited specifically by free fatty acids (FFAs) (29). Conflating the two generates incorrect mechanistic attributions.

### 3.6 Mechanosensory paresthesia

Tingling and numbing produced by hydroxy-α-sanshool (HAS) from Szechuan pepper engage mechanosensory pathways, with tingling principally attributed to inhibition of two-pore-domain potassium channels of the KCNK family (KCNK3, KCNK9, and KCNK18), generating abnormal tactile paresthesia without applied mechanical force (30, 31). These sensations are therefore grouped separately from the TRP-mediated categories: they share the trigeminal afferent route with pungency and cooling but not the receptor mechanism, and any TRPV1 or TRPA1 contribution at high hydroxy-α-sanshool concentrations is minor and uncertain (30).

### 3.7 Flavor modulation: kokumi

Kokumi modulates co-present taste attributes without contributing an independent taste quality when stimuli are presented in isolation. The proposed mechanism involves CaSR activation by γ-glutamyl peptides (32). CaSR involvement is supported, but its exclusivity, any basic-taste status, and its predictive value for food perception remain unresolved.

## 4 Molecular and chemical mechanisms

### 4.1 Polyphenol–protein interactions and astringency

Astringency is among the most intensively studied non-basic oral sensations, yet mechanistic consensus is only partial. It is useful to separate the proximal physicochemical events, which are well supported, from the downstream neural transduction, which is not resolved. The dominant framework attributes astringency to precipitation or conformational alteration of salivary proteins by dietary polyphenols, causing loss of the lubricating pellicle and increased friction perceived as dryness, roughness, or puckering (26, 27, 33). Proline-rich proteins (PRPs) are preferential polyphenol-binding partners, driven by hydrogen bonding and hydrophobic stacking (34). PRP proportion varies with saliva type, physiology, and sampling, introducing substantial inter-individual variability (35).

Three qualifications limit the protein-precipitation model. First, complexes do not inevitably precipitate; at sub-saturating concentrations, soluble complexes with altered lubrication properties are the proximal stimulus (27, 35). Second, mucins are central to boundary lubrication and are themselves polyphenol-binding substrates; however, the evidence that tannins deplete oral mucins is contested, with reports that tannins neither precipitated mucin nor removed significant mucin from the oral cavity, indicating that mucin loss alone is unlikely to account for friction-based sensation (36). Consistent with a mucin-protective role, an *in vitro* study using a custom-built biotribometer on reconstructed oral-epithelial models expressing different MUC1 isoforms found that epigallocatechin gallate (EGCG) disrupted epithelial lubrication and increased friction, while MUC1 expression and PRPs limited this loss, with PRPs binding tannins and reducing their interaction with mucins (37); this is *in vitro* epithelial tribology, not direct human sensory evidence. Third, the relationship between lubrication loss and perceived astringency is matrix-dependent and poorly predicted from single-compound binding assays (16, 26, 38). The temporal structure of astringency (onset delay, accumulation across samples, and carry-over) distinguishes it from equilibrium phenomena, and temporal dominance of sensations (TDS) characterizes this dynamic better than single-time-point ratings. Polyphenol structure is a critical determinant: degree of polymerization, galloylation, and flexibility modulate PRP binding and aggregation (39). Oxidized polyphenols formed during fermentation, ageing, or processing may show markedly different profiles, a complication that isolated-compound studies underestimate (40).

Astringency in plant-based protein matrices is especially complex. Legume proteins introduce astringency from residual phenolics, saponins, and oxidative browning products that differ from wine or tea astringency in composition and processing response (5). Recent evidence indicates that plant proteins may themselves contribute to astringency rather than acting only as carriers of residual phenolic compounds: sensory responses to isolated pea, lupin, and potato proteins were accompanied by salivary or mucin-related interactions and measurable haemodynamic (neural) responses, although these findings did not identify the peripheral receptor responsible for astringency transduction (6). A tribo-chemical analysis argues that effective prediction requires integrating the chemical reactivity of polyphenols with salivary proteins and the tribological consequences at the oral surface, dimensions usually studied in isolation (15). Consistent with recent syntheses, astringency is understood as a multimodal oral sensation involving interactions among astringent compounds, salivary proteins, the oral mucosal pellicle, lubrication loss, and changes in oral friction, such that sensory, biochemical, and tribological methods provide complementary rather than interchangeable information (41, 42). A further complication is that cell-wall biopolymers (pectins, galactomannans, arabinoxylans) modify pellicle properties and oral lubrication independently of polyphenol content, in ways sensitive to molecular architecture and interactions with salivary proteins (17). *In vitro* models calibrated on wine-type polyphenols therefore fail to predict plant-protein astringency, both because polyphenol composition differs and because the polymer matrix introduces lubrication variables outside the scope of precipitation-based assays. On the transduction side, emerging receptor-level evidence identifies TRPV2 and TRPC5 as possible compound-specific contributors to astringency; however, these findings are based mainly on heterologous expression systems and do not establish either channel as a dedicated human astringency receptor (43).

### 4.2 TRP channels and chemesthetic perception

Chemesthetic sensations are largely mediated by TRP channels on trigeminal afferents (25); the three channels considered in this review, their principal agonists, and their gating mechanisms are summarized in Figure 1. TRPV1 is activated by capsaicin at low micromolar concentrations through a well-characterized transmembrane binding site. Gingerols, shogaols and piperine also activate TRPV1 (19, 44). Desensitization explains heat adaptation during meals and is exploited in topical analgesics (45); lingual capsaicin pre-treatment can also transiently suppress responses to some tastants in humans (46). Chili matrices also contain constituents that suppress pungency: Borcherding et al. (11) identified chili-pepper compounds that reduced perceived pungency in sensory recombination tests; the molecular basis of the suppression was not established. Sensory pungency of a spice matrix therefore reflects the balance between activating and suppressing constituents, a complexity that single-compound studies miss.

**FIGURE 1:**
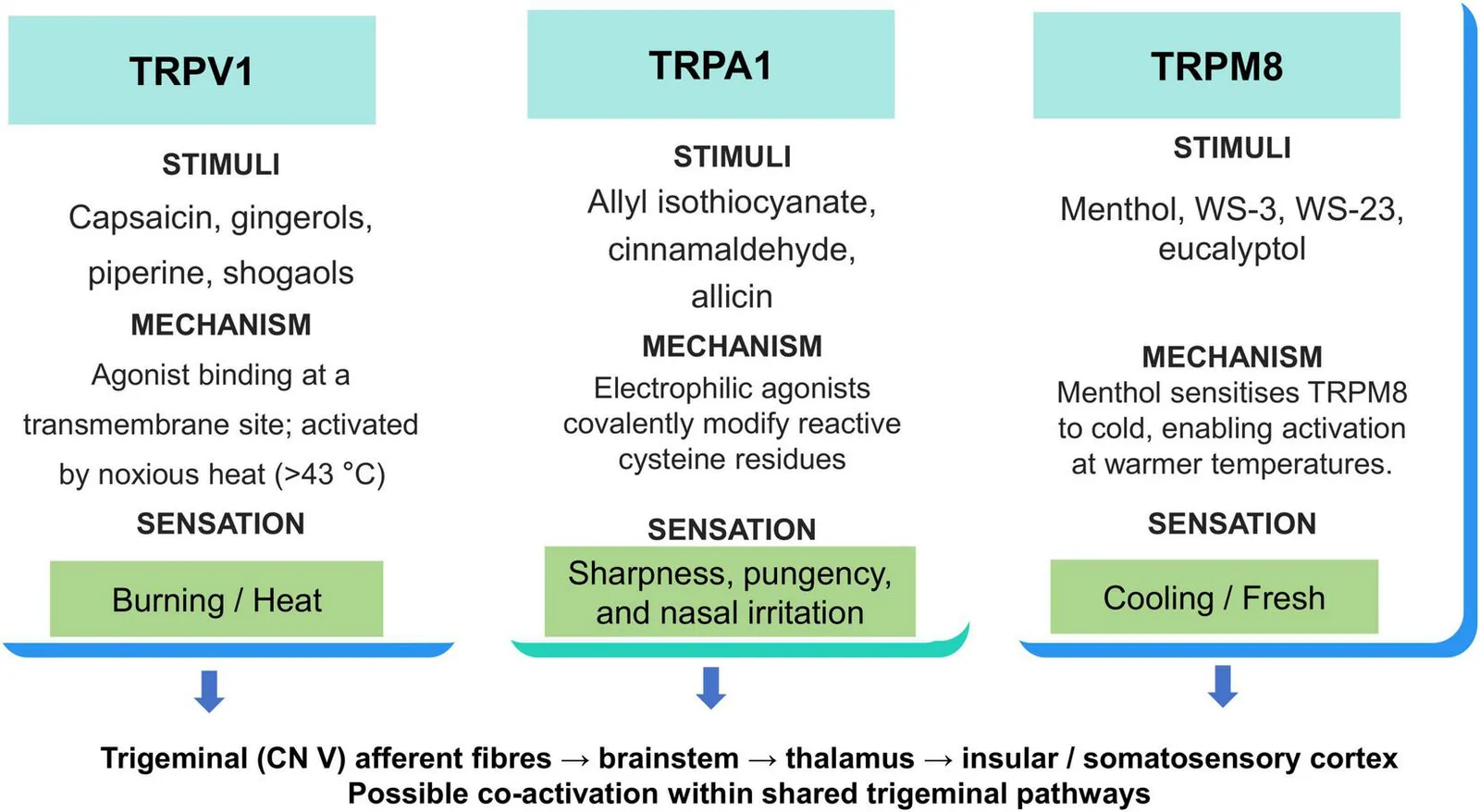
Conceptual overview of the three transient receptor potential (TRP) channels mediating oral chemesthesis in food systems. TRPV1 mediates capsaicin pungency and heat through a transmembrane binding site. TRPA1 mediates reactive pungency: electrophilic agonists activate it through covalent modification of reactive cytoplasmic cysteines, while non-electrophilic agonists may act differently. TRPM8 mediates cooling; menthol sensitizes the channel to cold, enabling activation at warmer temperatures. TRPA1 has been proposed to contribute to noxious cold responses, although its precise role in cold signaling remains debated (47). Where relevant, possible co-activation within shared trigeminal pathways is indicated; no direct receptor-to-receptor interaction is implied. Much of the receptor-level mechanistic characterization derives from heterologous expression systems and animal or *ex-vivo* models; translation to human food perception remains incompletely characterized. This figure focuses on TRPV1/TRPA1-mediated pungency and TRPM8-mediated cooling. Tingling and numbing are discussed separately in section “4.5 Tingling, numbing, and electric-like sensations”: KCNK channel inhibition contributes to sanshool-induced tingling (30, 31), whereas numbing-specific transduction remains unresolved. This is a conceptual diagram and does not represent experimental data. Supporting references: (19–22, 24, 44, 47, 49).

TRPA1 is activated by pungent electrophilic compounds; allyl isothiocyanate, cinnamaldehyde, allicin, and acrolein are among its food-relevant ligands, and it responds to a range of pungent compounds and bradykinin (21). The activating mechanism for electrophilic agonists is covalent modification of reactive intracellular cysteine residues, demonstrated by click-chemistry and mass-spectrometry labeling in a heterologous system (22); non-electrophilic agonists may act through different routes, so a single mechanism should not be assumed for all TRPA1 agonists. The resulting sensation (sharp, pungent, nasal-irritating) differs qualitatively from TRPV1 burning. TRPA1 has been proposed to contribute to noxious cold responses, although its role in oral cold signaling remains debated and is not treated as a primary mechanism in food chemesthesis (47). Receptor- and cell-level evidence identifies the activation mechanism but does not by itself predict food pungency intensity; food-compatible receptor assays may nonetheless provide useful prediction within defined product sets. A luminescence-based human TRPV1 assay reduced interference from food autofluorescence and showed an association with perceived pungency across six spicy noodle broths, although the limited product set, diluted samples, and sensory design restrict broader generalization (48).

TRPM8 mediates cooling sensations elicited by menthol and other cooling agents. Menthol sensitizes the channel to cold, allowing activation at warmer temperatures (20). A review of the molecular logic of cold sensation (47) describes TRPM8 as mediating innocuous cooling at sub-nociceptive intensities. More recent structural work has begun to characterize the conformational energetics of TRPM8 cold sensitivity (49), which may help rationalize differences in intrinsic receptor potency among cooling agonists. This structural perspective is promising but recent, and the relationship between receptor-level energetics and sensory performance in food depends additionally on matrix partitioning, salivary dilution, and mucoadhesion, and cannot be inferred from structural data alone.

The critical caveat for food applications is matrix effects on TRP activation. Capsaicin’s high lipophilicity causes preferential partitioning into fat phases and binding to hydrophobic protein sites, delaying mucosal contact and attenuating onset and peak heat. Consistent with this, a trained-panel study across model matrices and commercial salsas found that increasing starch, fat, and sugar content reduced perceived pungency (maximum intensity), with starch particularly influential and fat’s effect weaker in commercial salsas than in model systems (9). Such matrix effects mean that TRP-activation parameters measured in aqueous buffer poorly predict sensory pungency or cooling in food, a limitation underrepresented in the pharmacological literature.

### 4.3 Fatty mouthfeel and oleogustus: a necessary distinction

Fat contributes to oral sensation through mechanistically distinct pathways that must be separated. Fatty mouthfeel (creaminess, smoothness, richness, coating) arises from the physical properties of fat in the mouth and is a composite attribute spanning lipid-droplet behavior, salivary-protein interactions, mucin-film integrity, and mechanoreceptor engagement (14). Its dominant contribution operates through elastohydrodynamic lubrication: well-emulsified droplets reduce friction at the tongue–palate interface depending on droplet size, interfacial composition, and stability under shear (7, 8). The lubrication-based component of fatty mouthfeel does not require a taste-receptor mechanism; this does not exclude interactions with fatty-acid sensing or other sensory pathways. CD36 and GPR120 are not required to explain this lubrication-based component and are more relevant to fatty-acid sensing (oleogustus). Plant-derived biopolymers can partially replicate this lubrication through mucoadhesive film formation, though performance in the boundary regime is structure-dependent and generally does not match fat droplets at equivalent mass concentrations (17). Fatty mouthfeel is multidimensional–lubrication, viscosity, coating, emulsion breakdown, aroma release, saliva, and central integration–and tribology alone does not fully explain creaminess, richness, or the complete fat percept.

Oleogustus, proposed as an independent taste quality elicited by FFAs, is mechanistically distinct from fatty mouthfeel and should be treated separately. Human perceptual-mapping work indicates that medium- and long-chain nonesterified fatty acids have a taste sensation distinct from sweet, sour, salty, and bitter (with some overlap with umami), supporting perceptual distinctiveness and motivating the term oleogustus (29). Human oral-sensitivity data further implicate CD36: in 21 obese subjects, a common CD36 variant (rs1761667) associated with lower CD36 expression corresponded to about eight-fold higher oral detection thresholds for oleic acid and triolein, and a lipase inhibitor raised triolein but not oleic-acid thresholds, consistent with free fatty acid rather than triacylglycerol being the sensed stimulus (50). At the cellular level, work in cultured human fungiform taste-bud cells with siRNA knockdown indicates that CD36 responds to low fatty-acid concentrations while GPR120 responds only to high concentrations, and that GPR40/FFAR1 was undetectable in human (and rat) taste-receptor cells (51). These are human psychophysical and cultured-human-cell findings, respectively; neither establishes a sixth basic taste in whole-person psychophysics or in real foods. Several complications also remain: the sensory quality of FFAs at detectable concentrations is predominantly rancid and aversive; threshold FFA concentrations may exceed those of fresh food-grade fats; retronasal aroma from oxidation products is difficult to exclude; and whether CD36 and GPR120 function primarily as taste receptors or as post-ingestive satiety sensors is contested, given the established role of CD36 in fatty-acid-induced gut hormone release (52). A systematic review of the biological mediators of fat taste and smell concludes that CD36 and GPR120/FFAR4 are leading candidate mediators of oral fat chemosensation, but that fat perception also involves texture, olfaction, neural processing, genetic variability, and post-ingestive mechanisms; considerable variability in human fatty-acid sensitivity remains, and receptor-level evidence alone does not establish oleogustus as an independent basic taste (53). Consistent with a modulatory rather than autonomous role, human evidence suggests that GPR120 agonists may enhance fatty orosensation within an existing fat-containing system without independently evoking the sensation when presented alone (54). Oleogustus therefore remains a debated candidate taste quality rather than an established basic taste.

### 4.4 Metal-ion interactions and metallic sensation

Metallic sensation is among the most mechanistically ambiguous of these sensations, and no specific human metallic taste receptor has been identified. Metallic perception is not generated through a single dedicated pathway: metal salts may recruit gustatory, trigeminal, and retronasal olfactory mechanisms, with their relative contributions depending on the metal ion, associated anion, concentration, and pH, and although several receptors (including TRPV1, TRPA1, T1R3, and T2R7) have been proposed as candidate contributors, no specific human receptor dedicated exclusively to metallic perception has been established (55). Current evidence supports a multimodal account of metallic perception across metal species, with a particularly strong retronasal contribution for iron (12, 55). The best-evidenced pathway is catalytic production of volatile lipid-oxidation products: transition metals, especially iron, catalyze oxidation of polyunsaturated fatty acids in oral lipid films, generating metallic-smelling volatiles including 1-octen-3-one and 1-nonen-3-one (13). With nasal occlusion, FeSO_4_ metallic ratings fell to baseline in two experiments; comparable reductions were not observed for CuSO_4_ or ZnSO_4_ (12). Persistent metallic ratings for CuSO4 with the nose occluded in one experiment (12), together with evidence reviewed in (55), are the basis for cautiously retaining two minor contributions–direct trigeminal activation and possible weak gustatory interactions–neither of which is mechanistically resolved or should be described as receptor-mediated in the absence of an identified receptor. Because metallic perception is heterogeneous across metal species, lipid-oxidation volatiles should be treated as the principal account mainly for ferrous systems rather than for all metals. The absence of a validated instrumental proxy for metallic sensory intensity means iron-fortification formulation relies disproportionately on sensory panel feedback.

### 4.5 Tingling, numbing, and electric-like sensations

Hydroxy-α-sanshool (HAS), the primary alkylamide of Szechuan pepper, produces a buzzing, tingling, and ultimately numbing paresthesia qualitatively unlike capsaicin burning or menthol cooling (31). The mechanism involves inhibition of two-pore-domain potassium channels–KCNK3 (TASK1), KCNK9 (TASK3), and KCNK18 (TRESK)–on low-threshold mechanoreceptor neurones, depolarising them and generating abnormal tactile activation without applied mechanical force (30, 31). Any additional TRPA1 and TRPV1 contribution at high HAS concentrations is minor and uncertain. The numbing component is less securely resolved than the KCNK-mediated tingling mechanism; it is associated with KCNK modulation, but no specific numbing transduction pathway has been established. The combination in málà cuisine is a compelling case of concurrent multi-channel chemesthetic interaction. Food-derived numbing is, moreover, not restricted to sanshools or related alkylamides: evidence from pomelo peel identified coumarins as principal contributors to its numbing orosensation, indicating that chemically distinct food compounds may converge on similar perceptual outcomes (56). A key limitation is that HAS mechanism characterization has been conducted mainly in cell-based and *ex vivo* preparations, and food-matrix variables affecting bioavailability, stability, and delivery remain poorly characterized.

### 4.6 Kokumi and mouthfulness

Kokumi, first characterized by Ueda et al. (23), enhances and prolongs other taste attributes (sweetness, saltiness, umami) while conferring thickness, body, and continuity, without contributing an independent taste quality in isolation (23, 32). The molecular basis is usually attributed to γ-glutamyl peptides, particularly γ-Glu-Val-Gly and glutathione, proposed to activate CaSR in type II taste-bud cells through paracrine signaling (32, 57). The evidence supports CaSR involvement in taste enhancement, but three qualifications apply. First, demonstrating that CaSR is expressed in taste tissue and activated by γ-glutamyl peptides *in vitro* does not establish that CaSR activation causes the kokumi percept in humans, and the concentrations required for CaSR activation *in vitro* can exceed those in the fermented foods associated with kokumi (57). Ohsu et al. additionally provided human antagonist evidence supporting CaSR involvement in model solutions (32). Second, γ-glutamyl peptides may act through broader taste-interaction routes rather than a single dedicated receptor; a recent proposal identifying novel kokumi peptides from yeast extract rests on computational (*in silico*) docking rather than direct receptor demonstration and should be read as suggestive (58). Third, kokumi may be a composite flavor-enhancement percept rather than a discrete single-mechanism sensation. Real-food evidence does exist: in chicken consommé, addition of the kokumi peptide γ-Glu-Val-Gly significantly increased “umami,” “mouthfulness,” and “mouth-coating” relative to controls in a descriptive analysis with 18 trained female panelists (59).

Recent work reinforces this reading. A recent review (several of whose authors are affiliated with a commercial food-ingredient manufacturer, warranting cautious interpretation) classifies kokumi as a taste-related oral perception produced by taste modulators rather than an independent basic taste: kokumi substances are generally weak or tasteless alone but may enhance thickness, mouthfulness, continuity, richness, complexity, and lastingness in the presence of other taste stimuli, with CaSR the best-characterized candidate pathway although other receptors and central processes may contribute (60). At the structural level, cryo-electron microscopy and receptor mutagenesis have strengthened the molecular basis of γ-glutamyl peptide recognition by CaSR, yet structural binding and receptor activation do not establish that CaSR activation alone is sufficient to generate kokumi perception in humans or in finished foods (61). Food-derived studies show the same gap between receptor activity and confirmed perception: enzymatic treatment of dry-cured lamb crust generated multiple γ-glutamyl peptides and increased CaSR activation, and glutathione-derived Maillard reaction products altered CaSR activation in a sugar- and temperature-dependent manner (with glucose-derived products at elevated temperatures producing stronger responses than several xylose-derived conditions, and with browning and volatile generation not directly predicting activation), but neither study included human sensory or finished-food validation (62, 63). Kokumi thus has practical relevance for restoring flavor complexity in reduced-ingredient formulations; its mechanistic confidence is MODERATE because CaSR involvement is supported by molecular, structural and human antagonist evidence in model solutions (32), while its exclusivity, sufficiency, and predictive value for food matrices remain unresolved.

## 5 Neurophysiology and multimodal perception

### 5.1 Trigeminal architecture and oral somatosensation

The trigeminal nerve (CN V) provides the primary somatosensory innervation of the oral cavity through its maxillary and mandibular branches, carrying signals from mechanical deformation, temperature change, and chemical irritation to the trigeminal nucleus caudalis and then to the thalamus and cortex (24, 25). This pathway converges with gustatory signals from CN VII and CN IX at the thalamus and insular cortex, where multimodal flavor integration occurs (1, 3, 25). Functionally, cooling responses tend to adapt while capsaicin-evoked heat can sensitize with repeated sub-maximal stimulation (24, 45)–dynamics not captured by single-measurement ratings, which require time-intensity or temporal-dominance methods (64).

### 5.2 Cross-modal interactions and multimodal integration

Flavor perception is a multisensory achievement rather than the summation of independent gustatory signals; olfactory, gustatory, trigeminal, and somatosensory inputs interact peripherally and centrally (in the insular and orbitofrontal cortex) to produce percepts not predictable from any single modality (3). These interactions are frequently non-additive and directionally asymmetric: concurrent stimuli can enhance or suppress each other depending on relative concentrations, temporal alignment, and the attributes involved (4). Figure 2 presents an integrative conceptual model of these interactions, distinguishing well-evidenced pathways from proposed or contested ones. The neural substrate for cross-modal integration has been characterized in the orbitofrontal cortex, where convergent gustatory, olfactory, and somatosensory projections form unified hedonic representations (1, 65). This representation is experience-dependent, consistent with the role of cultural exposure and dietary history in hedonic responses to capsaicin, polyphenol astringency, and kokumi. Direct human neuroimaging supports early cortical convergence: in a preregistered fMRI study of 25 participants, taste-associated retronasal odors and their corresponding tastes produced overlapping flavor-specific representations, particularly in dysgranular and agranular regions of the anterior insula, supporting the insula as a possible early flavor-integration hub–though this does not establish identical integration mechanisms for every non-basic oral sensation (66).

**FIGURE 2:**
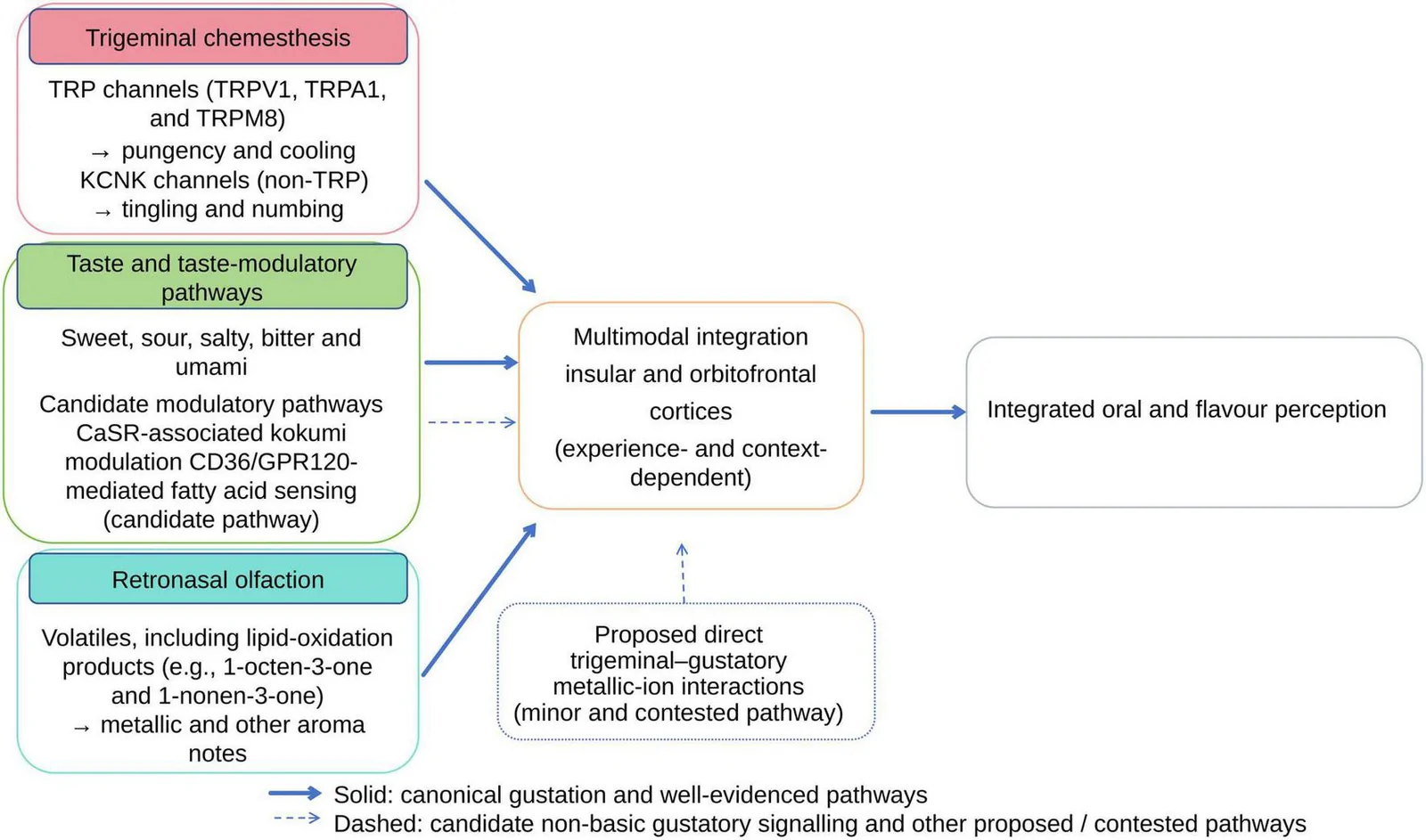
Integrative conceptual model of non-basic oral perception. Solid arrows indicate well-evidenced pathways; a solid arrow indicates canonical gustation, while dashed arrows indicate candidate non-basic gustatory signaling and other proposed or contested pathways (CaSR-mediated kokumi; candidate CD36/GPR120 free-fatty-acid sensing; metallic-ion–epithelial interaction). CD36 and GPR120 appear in this model in relation to oleogustus, that is, free-fatty-acid sensing, and not to the lubrication-based component of fatty mouthfeel, which is mechanoreceptor-mediated. Within the trigeminal chemesthesis node, TRP-mediated pungency and cooling are shown separately from the KCNK-associated grouping of tingling and numbing. This grouping is schematic and does not imply equally established mechanisms: KCNK channel inhibition contributes to sanshool-induced tingling (30, 31), whereas numbing-specific transduction remains unresolved. The grouping denotes a shared trigeminal afferent pathway, not a shared receptor family. The model emphasizes that no single sensory pathway accounts for the full range of non-basic oral sensations, and that trigeminal, gustatory, and retronasal olfactory signals converge in cortical integration to generate the integrated oral and flavor percept. This is a conceptual diagram and does not represent experimental data. Supporting references: (1, 3, 12, 25, 32, 65).

Specific cross-modal interactions relevant here include the following. Pungency–saltiness: sub-threshold capsaicin has been proposed to enhance perceived saltiness through central cross-modal enhancement (67), a possible basis for sodium reduction that requires replication across matrices; more broadly, TRPV1- and TRPA1-mediated chemesthesis may modify selected taste responses through peripheral trigeminal–gustatory interactions, neuropeptide signaling, and central integration, though these effects are stimulus- and context-dependent and human findings are not uniform (68). Astringency–fat: dietary fat attenuates polyphenol-induced astringency; the lubrication contribution is well supported, whereas any specific competitive lipid-binding mechanism is less directly evidenced and should be distinguished from the lubrication effect (38). Astringency–aroma: sensory evaluation of wine phenolic fractions indicates that added aroma compounds can modify perceived bitterness and astringency sub-qualities, so cross-modal influences on astringency are not limited to lubrication effects (69). Fat–aroma: fat modulates the timing and intensity of retronasal aroma through partitioning of volatiles between lipid and aqueous phases in ways not predictable from equilibrium partition coefficients alone (14).

### 5.3 Individual differences

Inter-individual variability is large, often comparable to between-compound differences. Sources include salivary flow and protein composition (35), cultural exposure and acquired expertise, which are associated with taste phenotype (70), and oral-microbiome composition (71). The oral microbiome is an active participant: resident microbiota metabolize polyphenols, contribute to volatile generation, and may influence mucin turnover, so two individuals may experience different astringency, metallic, or aroma-linked sensations partly for microbial reasons (71). Salivary PRP output is the primary modifier of polyphenol-mediated astringency, with higher output associated with lower perceived astringency (35). Ageing is a particularly relevant axis: older adults often show reduced salivary flow (72) and may show altered TRP-channel and tactile responsiveness, shifting perception of pungency, cooling, and fat-related mouthfeel–precisely the population targeted by fortified and texture-modified foods. Variability in oral fat sensitivity (50) and oral-processing behavior (73) further affects fatty mouthfeel and oleogustus. Consequently, trained-panel data, which exclude population variability by design, may fail to predict consumer acceptance for sensations with large genetic, physiological, age-related, or experiential variability.

## 6 Food matrix effects and processing influences

The food matrix is an active participant in the generation, modulation, and suppression of non-basic oral sensations, a principle underrepresented in studies that characterize compounds in aqueous solution and extrapolate to food (7, 9, 15, 17, 28). Table 4 summarizes the principal food-matrix factors discussed in this section and their implications for perception.

**TABLE 4:** Food-matrix factors influencing non-basic oral sensations and their implications for sensory perception.

Sensation	Representative food examples	Key compounds/structures	Matrix factors affecting perception	Practical implication
Astringency	Tea, wine, plant-based proteins	Polyphenols/tannins interacting with salivary proteins, especially proline-rich proteins (PRPs) (26, 27, 33, 34)	Salivary composition, protein–polyphenol interactions, oral lubrication loss, frictional changes, and food matrix structure influence perceived astringency (15, 17, 28, 35)	Reducing astringency requires consideration of chemistry and oral tribology rather than polyphenol concentration alone (15, 26)
Pungency	Chili products, spicy foods	Capsaicin and other pungent compounds activating TRPV1/TRPA1 pathways (19, 21, 22)	Fat, starch, sugar, partitioning, and interactions among activating and suppressing compounds alter perceived pungency (9–11)	Formulation strategies should consider compound delivery and matrix effects rather than receptor activity alone (9, 11)
Cooling	Mint products, cooling agents	Menthol and synthetic cooling compounds acting through TRPM8 (20, 47)	Effects of food-matrix composition, release kinetics, and oral processing on cooling delivery and persistence require direct validation in finished foods; biosensor-sensory comparisons used simplified solutions (81)	TRPM8 biosensor responses may support screening, but predictive validity for cooling in finished foods was not assessed (81)
Fatty mouthfeel	Dairy foods, emulsified foods, fat-reduced products	Lipid droplets, interfacial structures, lubrication mechanisms, and oral film formation (7, 8, 14)	Droplet size, emulsion stability, viscosity, and lubrication behavior influence creaminess and smoothness (7, 8, 14)	Fat replacement requires control of physical structure, not only lipid content reduction (7, 8)
Metallic sensation	Iron-fortified foods, mineral-containing foods	Metal-catalyzed lipid oxidation products, especially volatile oxidation compounds from iron systems (12, 13)	Metal species, lipid composition, pH, and retronasal aroma contribution influence metallic perception (12, 13, 55)	Sensory evaluation remains essential because no validated universal instrumental predictor exists (12)
Oleogustus	Fat-containing foods, fatty acid stimuli	Free fatty acids and candidate receptors including CD36 and GPR120/FFAR4 (29, 50, 51)	Fatty acid concentration, oxidation, texture, and interaction with other sensory pathways influence perception (53)	Oleogustus should be considered a proposed taste-related quality rather than an established basic taste (53)
Tingling/numbing	Sichuan pepper and málà cuisine	Hydroxy-α-sanshool and KCNK channel modulation (30, 31)	Compound delivery, concentration, and food matrix effects influence chemesthetic intensity (30, 31)	Mechanistic understanding remains stronger than prediction in real foods (30, 31)
Kokumi	Fermented foods, broths	γ-Glutamyl peptides and proposed CaSR activation (23, 32, 57)	Interactions with existing taste stimuli and food composition determine enhancement effects (32, 59)	Kokumi is best interpreted as a flavor-modulating phenomenon rather than an independent basic taste (32, 60)

### 6.1 pH and beverage-matrix composition

Polyphenol-mediated astringency is sensitive to matrix pH: in wines, cranberry juice and model phenolic solutions, lower pH has generally been associated with greater perceived astringency, an effect attributed to a reduced proportion of charged phenolate ions and consequently greater potential for hydrogen bonding between polyphenols and salivary proteins (74). Wine polysaccharides also modulate salivary protein–tannin interaction and hence the precipitation step (39). This complexity explains why wine astringency cannot be predicted from total polyphenol content alone (16, 40).

### 6.2 Protein and fat matrix effects

Lipophilic sensory-active compounds distribute between aqueous and lipid phases, and only the freely available fraction reaches its receptor (9, 14); the capsaicin partitioning effect of section “4.2 TRP channels and chemesthetic perception” is one instance. The same logic governs astringency masking by fat and protein: milk proteins compete with salivary PRPs as polyphenol-binding substrates, reducing the polyphenol fraction available to precipitate salivary proteins–the basis of dairy-mediated astringency masking in tea and wine (74). In each case the bulk concentration is a poor predictor of perceived intensity because the matrix determines sensory availability.

### 6.3 Processing effects

Processing can also modulate plant-protein astringency: empirical and anecdotal evidence suggests that fermentation can reduce astringency, although the accompanying fall in pH may itself affect astringency, and Maillard conjugation of plant proteins with polysaccharides has reduced friction in tribological measurements (5). Fat-droplet characteristics influence the rheological, tribological and sensory properties of emulsion-filled food gels (7), while particle-stabilized (Pickering) emulsions provide an alternative approach to controlling emulsion interfacial structure and stability in food applications (75).

### 6.4 Plant-based foods as a matrix-complexity case study

Plant-based foods provide a useful example of matrix-mediated modification. In plant-protein systems, protein source, phenolic composition, microstructure, and saliva-mediated lubrication can influence astringency and mouthfeel (5, 6, 15, 17). Extruded plant-based meat alternatives present additional flavor challenges arising from ingredient composition and processing, and flavor optimisation remains a recognized challenge in this product category (76). Together, these examples illustrate the importance of matrix and processing context when interpreting sensory behavior in plant-based foods. Because this review was targeted rather than exhaustive (section “2.1 Databases and search strategy”), this interpretation reflects the evidence identified here rather than a field-wide survey of predictive tools; artificial-intelligence methods are increasingly applied to sensory prediction, as discussed in section “10 Future perspectives and research directions.”

## 7 Analytical and sensory measurement techniques

Table 5 summarizes the principal sensory, instrumental, and emerging measurement techniques discussed in this section, together with their applications, strengths, and main limitations.

**TABLE 5:** Measurement techniques for non-basic oral sensations: applications, strengths, weaknesses, and best-suited sensations.

Method	Application	Strengths	Key weaknesses	Best-suited sensations
QDA – Quantitative descriptive analysis	Trained panel, static intensity ratings	Reproducible; detailed profiling; between-product comparison	Misses temporal dynamics; training-intensive; unrepresentative of population variability	Astringency magnitude, metallic intensity, fatty mouthfeel
Time-intensity (TI)	Single attribute recorded continuously	Captures onset, peak, persistence, carry-over	One attribute per session; training burden	Astringency, pungency, cooling, tingling
Temporal dominance of sensations (TDS)	Multiattribute temporal dominance	Multiple attributes at once; ecologically valid	Dominance ≠ intensity; statistical complexity; lexicon pre-defined	Wine, spiced sauces, plant-based beverages
CATA / TCATA	Consumer descriptive profiling	Low training burden; large samples; rapid screening	No intensity information; pre-defined lexicon	Screening, consumer research, kokumi detection
Oral tribology (Stribeck)	Friction of food–saliva films	Mechanistically direct; correlates with mouthfeel and astringency	Model surfaces approximate oral biology; saliva variability	Astringency, fatty mouthfeel, fat-replacer evaluation
Rheology (oscillatory, flow)	Viscosity, viscoelasticity, gel strength	Instrumental, reproducible; links bulk texture to mouthfeel	Misses tribological regime; poor for thin liquids	Fat-related texture, hydrocolloid mouthfeel
GC-MS / GC-olfactometry	Volatile identification and odor activity	High sensitivity; links chemistry to aroma	Static headspace ≠ retronasal dynamics; extraction artifacts	Metallic sensation, pungent volatiles, off-flavor
LC-HRMS / HPLC	Polyphenol, peptide, fatty-acid quantification	High accuracy and selectivity; broad coverage	Bound vs. free may be indistinguishable; prep may alter matrix	Astringency, kokumi peptides, oleogustus FFAs
ITC / fluorescence quenching	Binding thermodynamics and stoichiometry	Thermodynamic parameters; mechanistic insight	Solution phase only; salivary complexity not replicated	Astringency mechanism research; not food prediction
fMRI / EEG	Cortical processing and integration	Direct neural evidence; maps integration sites	Incompatible with complex foods; cost; limited temporal resolution (fMRI)	Fundamental neuroscience; individual differences
Cell-based receptor assays	Receptor dose-response, EC_50_ ranking	Mechanistic specificity; high throughput	No matrix context; no pharmacokinetics; expression artifacts	Mechanistic research; NOT food sensory prediction
TRPM8 cell-based cooling biosensor (emerging)	*In vitro* cooling quantification; coolant-mixture interaction analysis (Chou–Talalay)	Human TRPM8 readout; quantifies synergy/antagonism; correlates with trained panel	Simplified solutions ≠ finished foods; cell-based, lacks full oral context	Cooling-agent screening and formulation
AI / QSAR modeling	Structure-to-sensory prediction	Scalable; integrates large databases	Low accuracy for complex matrices; purified-compound training; poor generalizability	Exploratory research; hypothesis generation

TI, time-intensity; TDS, temporal dominance of sensations; CATA, check-all-that-apply; ITC, isothermal titration calorimetry; LC-HRMS, Liquid Chromatography High-Resolution Mass Spectrometry; QSAR, quantitative structure–activity relationship; GC-O, gas chromatography–olfactometry; FFA, free fatty acid.

### 7.1 Sensory methods

Quantitative descriptive analysis (QDA) with trained panelists remains the benchmark for sensory profiling, but its reliance on single-time-point ratings makes it inadequate for sensations with distinctive onset, build, and persistence (64, 77). Time-intensity (TI) improves resolution of dynamic sensations and has been applied to astringency, cooling, and pungency (64). Temporal Dominance of Sensations (TDS) captures which attribute dominates at each time point across simultaneously perceived qualities (64). Check-all-that-apply (CATA) questions generate lexicon-based profiles without the training burden of QDA (77), and their data can be analysed with established statistical approaches (78).

### 7.2 Instrumental and physicochemical methods

Oral tribology–friction between model surfaces lubricated by food–saliva mixtures as a function of sliding speed–has become a key tool for mouthfeel and astringency (8). Stribeck curves reveal the lubrication regime; polyphenols shift measurements toward boundary lubrication, while fat and mucins shift them toward hydrodynamic lubrication (7, 17, 26). For volatile-mediated sensations, GC-MS with GC-olfactometry provides compound identification and perceptual relevance (13). LC-MS-based methods, including LC-HRMS, are used to characterize non-volatile phenolic compounds and lipid-degradation products such as free fatty acids (16, 76). Isothermal titration calorimetry (ITC) and fluorescence quenching determine thermodynamic and kinetic parameters of polyphenol–protein interactions (35).

### 7.3 Neurophysiological and emerging methods

EEG and fMRI have been applied to map cortical processing of chemesthetic, texture, and flavor-integration signals (79, 80). The orbitofrontal cortex is characterized as a primary site of multimodal flavor integration (1, 65). Cell-based receptor assays (TRPV1, TRPA1, TRPM8, CaSR) provide mechanistic specificity and throughput (19, 21, 32)– the CaSR assays underpinning the kokumi literature are one example (32) – but they operate without oral-matrix complexity or pharmacokinetic context (9, 15). As an emerging measurement approach, a human TRPM8 cell-based biosensor combined with Chou–Talalay analysis has been used to quantify concentration- and ratio-dependent interactions among menthol, WS-3, and WS-23; its correspondence with trained-panel ratings supports its value as a formulation-screening method, although testing in simplified solutions does not establish transferability across complex finished foods (81). Artificial-intelligence approaches offer scalability for sensory prediction but currently lack accuracy for complex food matrices (section “10 Future perspectives and research directions”).

## 8 Industrial applications and product design

Table 6 summarizes the industrial strategies discussed in this section, presenting mechanistic status separately from practical actionability.

**TABLE 6:** Industrial strategies for modulating non-basic oral sensations, with mechanistic status shown separately from practical application.

Problem/sensation	Formulation strategy	Mechanistic basis	Mechanistic status	Food application	Trade-offs/limitations
Polyphenol astringency (plant-protein beverages)	Tannase; polysaccharide addition; dairy-protein co-formulation; fermentation	Polyphenol hydrolysis; competitive PRP binding; steric stabilization; adduct modification (15, 82, 86, 88)	Established for tannase chemistry and polysaccharide–protein interactions; product-specific for finished-food sensory outcomes; direct evidence for dairy-protein co-formulation and fermentation was not identified in this review	Pea/oat protein beverages; legume extracts	Tannase alters polyphenol composition and may affect flavor; increased antioxidant activity has been reported in green tea (87), while reduced astringency has been reported in pomegranate juice (88). Polysaccharide addition may increase viscosity (17); dairy precludes vegan positioning; fermentation may alter flavor.
Loss of creaminess in reduced-fat dairy	Protein microgels; oleogels; modified starch; beta-glucan; designed emulsifiers	Restoration of boundary lubrication; viscosity matching; droplet-interface engineering (7, 8, 84, 85)	Established for the lubrication mechanism; product-specific to experimental for individual replacers (oleogels, protein microgels)	Low-fat yoghurt, reduced-fat cheese, dairy beverages	Each replicates only a subset of fat functions; some polyelectrolytes may increase dryness (17).
Metallic off-flavor in iron-fortified foods	Lipid encapsulation; ferrous bisglycinate; antioxidant co-addition	Intended to reduce free-Fe^2+^–lipid contact, thereby limiting retronasal volatile generation (12, 13, 89)	Product-specific: demonstrated for lipid oxidation in microencapsulated iron-fortified drink yogurt (89)	Iron-fortified beverages and foods	No instrumental proxy; universal removal unproven. Bioavailability was not assessed in the cited study; generalization across encapsulants, iron sources and food matrices remains unestablished.
Excessive capsaicin heat	Microencapsulation; capsaicinoid selection; variety selection	Delayed TRPV1 access; extended release; balance of activating/suppressing constituents (10, 11, 19)	Product-specific for encapsulation (demonstrated in one nanocapsule system); experimental for the suppressor-compound route	Chili sauces, functional foods, spiced snacks	Encapsulation alters release timing and is formulation-specific (10); variety and processing may influence the retention of suppressor relative to activator compounds, although this was not directly tested (11).
Loss of richness in plant-based meat	Yeast extract; fermented soy; matured vinegar; γ-Glu peptides	Possible CaSR-mediated enhancement and/or broader peptide taste interactions (32, 57, 58, 60)	Experimental: human pharmacological evidence supports CaSR involvement (32); generalizability across complex food matrices remains insufficiently established; several newer peptides supported only by computational or matrix-specific evidence (58, 60)	Plant-based patties, sausages, ready meals	Reported as effective in specific matrices, for example enhancement of mouthfulness in chicken consommé by γ-glutamyl-valyl-glycine (59), but the mechanism remains uncertain and each product requires separate validation.
Cooling and freshness delivery	Cooling-compound selection (menthol, WS-3, WS-23)	TRPM8 activation; menthol sensitizes the channel to cold, enabling activation at warmer temperatures (20, 24)	Established receptor mechanism; product-specific performance requiring food-specific human validation	Chewing gum, beverages, oral care, confections	Synthetic-agent performance, odor, and stability are matrix-dependent; effects on other attributes require validation.
Astringency carry-over across samples	Dairy protein; mucoadhesive polysaccharide coating; polyphenol reduction	Competitive PRP binding; mucin protection; reduction of reactive polyphenol (15, 17, 82)	Established mechanism; product-specific magnitude, individual-dependent	Polyphenol-rich beverages; ready-to-drink tea/coffee	Dairy precludes vegan positioning; carry-over varies with salivary protein output (35) and individual astringency responsiveness (90).

PRP, proline-rich protein; CaSR, calcium-sensing receptor. Mechanistic status is reported separately from practical application and formulation limitations so that empirically useful strategies are not presented as mechanistically proven. All strategies require product-specific sensory validation, and reported effects should not be assumed to generalize across formulations or processing conditions. Representative supporting studies are cited where relevant.

### 8.1 Astringency management in plant-based and functional foods

Polyphenol- and protein-associated astringency is commercially important in plant-protein beverages and other phenolic-rich foods (5, 6, 15). Potential mitigation approaches include modifying phenolic or protein structure, controlling aggregation and particle properties, and using ingredients that alter salivary-protein interactions or oral lubrication (5, 15, 17). Yeast mannoproteins may modulate polyphenol-related astringency by changing flavanol–salivary-protein interactions. In a model system, a carbohydrate-rich mannoprotein promoted soluble ternary aggregates, whereas a mannoprotein with a higher protein proportion precipitated mainly non-galloylated flavanols and partially reduced insoluble flavanol–salivary-protein aggregation. These mechanistic findings require direct sensory confirmation in finished foods (5, 15, 82). Practical use may also involve product-specific trade-offs related to flavor, formulation complexity, cost, and other quality attributes (76).

A tribo-chemical perspective suggests that mitigation effectiveness should be assessed by both chemical consequences (reduction of reactive polyphenol or competitive PRP binding) and tribological consequences (whether the intervention restores lubrication at the oral surface) (15). This is especially relevant for polysaccharide strategies, where the tribological benefit of film formation may be as important as, and mechanistically independent of, steric stabilization (17). Supporting the practical value of such measurements, a study of acidic plant-protein-fortified fiber-based smoothies found that boundary friction, rheological properties, and protein insolubility were associated with trained-panel ratings of mouthfeel and astringency, supporting tribology as a formulation-screening tool–albeit one whose predictions depend on protein source and acidic beverage conditions and so generalize only cautiously (83). The oral lubrication performance of individual plant-derived polymers (pectins, galactomannans, arabinoxylans) is beginning to be characterized as a function of molecular structure and polymer–saliva interactions, offering a more rational basis for selection than empirical screening, though this literature is recent (17).

### 8.2 Fat reduction and texture engineering

Fat reduction is the most mechanistically complex challenge because fat simultaneously contributes tribological lubrication, viscosity, and flavor-release dynamics (7, 8, 14). Rational fat-replacer design requires specificity about which mouthfeel attributes consumers associate with richness and their mechanisms: not all richness is tribological, and coating, after-feel, and flavor persistence may require distinct replacement strategies (14).

Simple hydrocolloid-based approaches to fat reduction have not achieved the required consumer acceptability, and low sliding friction has been proposed as a target for fat mimetics (1); texture perception of gelled and emulsion-filled foods also depends on how their microstructure breaks down during oral processing (84). Structured-oil systems such as oleogels offer tribological properties more similar to solid fat (85). Protein microgels can act as fat mimetics by providing smooth, deformable particles engaging lubrication mechanisms similar to fat droplets (7, 8). Plant-derived biopolymers offer an additional route through mucoadhesive film formation; however, polymers that compete with salivary mucins may paradoxically increase perceived dryness, as reported for some highly charged polyelectrolytes under physiological salivary conditions (17). Selection requires oral-tribology data across the relevant sliding speeds, not rheology alone (7, 8).

### 8.3 Chemesthetic design: controlled pungency and cooling

Controlled pungency is pursued through capsaicin microencapsulation in lipid or protein matrices, delaying mucosal contact and extending the temporal profile, or through capsaicinoid analogues with different TRPV1 kinetics (9, 10, 19). Encapsulation effects are formulation-specific rather than general: in a controlled study of capsaicin-loaded core–shell nanocapsules, nanoencapsulation significantly attenuated perceived pungency in a trained-panel evaluation, whereas a chitosan shell did not mask pungency (10); such results should not be generalized across encapsulation systems. An emerging complementary route is the endogenous chemical complexity of spice matrices: Borcherding et al. (11) identified chili-pepper compounds that suppress pungency, suggesting that variety selection and processing conditions influencing the retention of suppressor versus activator compounds may modulate pungency.

Cooling-compound selection has become more sophisticated with growing understanding of TRPM8 (20, 49). Recent structural work on TRPM8 cold sensitivity (49) may help rationalize differences in intrinsic receptor potency but should not, on its own, support commercial claims about food performance. Synthetic cooling agents such as WS-3, WS-23, and menthol differ in their sensory performance, but these differences are matrix-dependent and cannot be predicted from TRPM8 discovery or structural data alone; the performance of any specific coolant in a specific food matrix requires product-specific sensory validation (81).

### 8.4 Functional foods, iron fortification, and plant-based Foods

The expanding plant-based market compounds these challenges: legume proteins introduce astringency from saponins and residual phenolics plus beany off-flavors from lipoxygenase-derived volatiles (5, 6), managed through tannase/polyphenol-oxidase treatment, fermentation, and particle-size modification (5, 86). Tannase hydrolyses galloylated polyphenols, modifying their interactions with proteins and improving the sensory attributes of green tea infusion (86); tannase treatment has also been reported to increase antioxidant activity in green tea (87) and reduce astringency in pomegranate juice (88). Iron fortification of plant-based milks, cereals, beverages, and infant formulas is chronically limited by metallic off-flavors from ferrous sulfate (12, 13); strategies such as microencapsulation in lipid matrices or organic iron sources aim to reduce free-metal contact with oral epithelium and lipid-oxidation substrates (12, 89). Direct product-specific evidence supports this approach in at least one system: in microencapsulated iron-fortified drink yoghurt, lipid oxidation (thiobarbituric-acid values) was significantly lower in encapsulated than uncapsulated treatments during storage (89). These effects are product-specific; encapsulation or organic iron sources such as ferrous bisglycinate should not be assumed to eliminate metallic flavor or astringency universally in the absence of a direct food sensory study. Kokumi-active peptides from fermented ingredients have potential application in reduced-ingredient and plant-based formulations; however, evidence for several newer peptides remains computational or matrix-specific and requires direct product validation (58, 60).

### 8.5 Cross-sensation strategies for sodium and sugar reduction

Non-basic oral sensations offer mechanistically grounded routes to sodium and sugar reduction, each requiring matrix-specific validation (60, 67). Sub-threshold capsaicin has been proposed to enhance saltiness (67), potentially permitting sodium reduction without proportional loss of saltiness, an effect requiring sensory validation in specific matrices (67, 68). Cooling compounds are useful for delivering cooling and freshness; their performance is matrix-dependent and requires product-specific human sensory validation (81). Kokumi-enhancing peptides can restore flavor roundness lost when salt, fat, or sugar are reduced (60).

## 9 Discussion: challenges, controversies, and knowledge gaps

The two-axis assessment introduced in section “2.3 Evidence assessment: two distinct axes” is summarized in Figure 3, which positions the ten mechanistic entries by mechanistic confidence and food-matrix translation confidence. The distribution is informative: no entry reaches high confidence on both axes, and every entry rated LOW on translation also falls short of HIGH on mechanism, indicating that secure mechanistic identification alone has not been sufficient to achieve high confidence in food-matrix translation within the evaluated sensations. The sections below examine the principal challenges underlying that gap.

**FIGURE 3:**
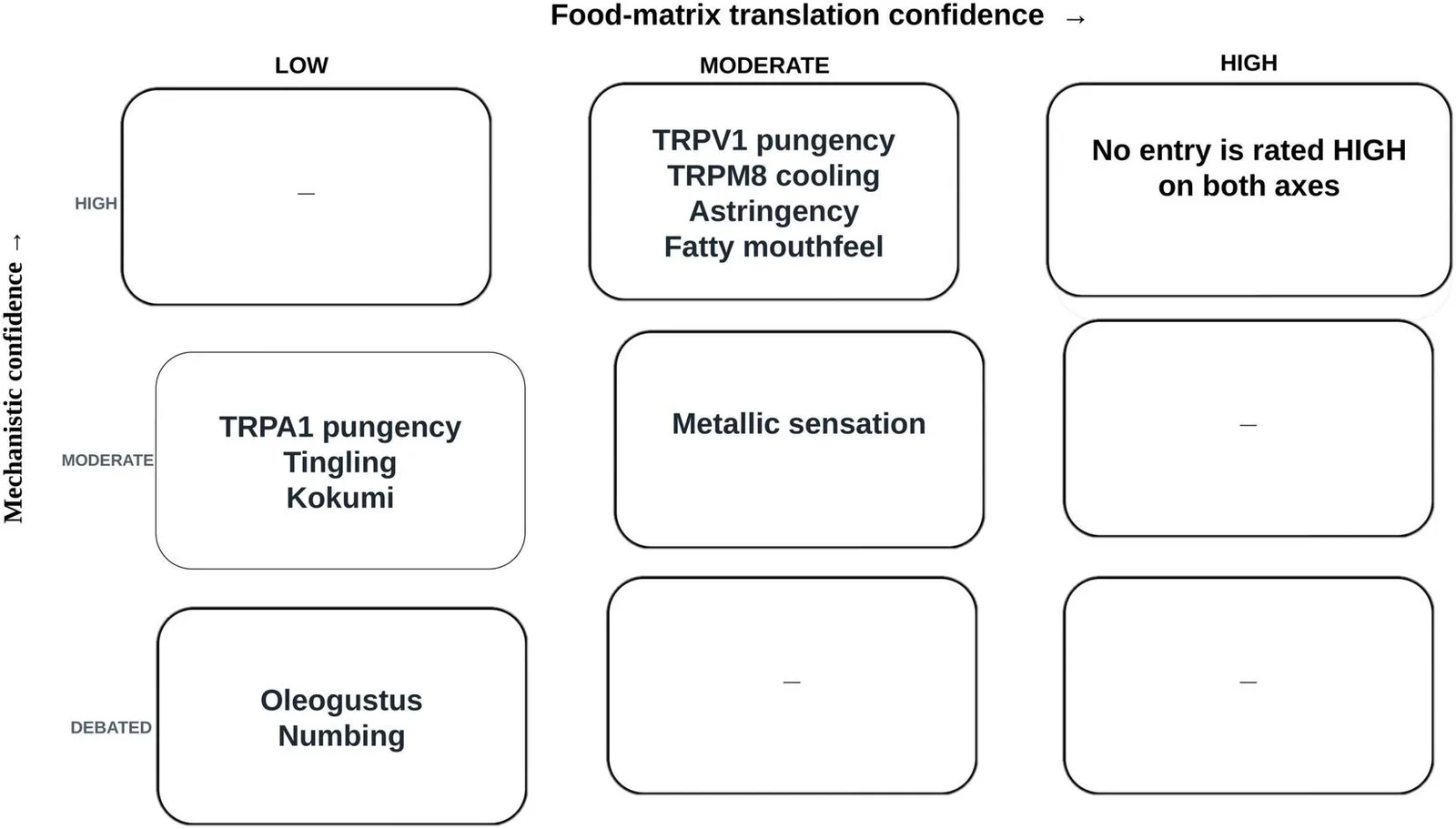
Qualitative evidence matrix positioning ten mechanistic sensation/pathway entries derived from the nine target non-basic oral sensations according to confidence in their principal proximal mechanisms and translation to real-food matrices. Both axes are labeled explicitly in the figure as ordinal categories: the horizontal axis, translation confidence, is divided into LOW, MODERATE and HIGH columns, and the vertical axis, mechanistic confidence, into DEBATED, MODERATE and HIGH rows. Each entry is placed in the cell corresponding to its two ratings in Table 2, so the figure and Table 2 can be read against one another directly. Entries fall along a diagonal band rather than filling the matrix: the upper-right cell, denoting high confidence on both axes, contains no entry, nor do the high-mechanistic/low-translation and debated/moderate cells. The two axes therefore capture distinct but qualitatively related dimensions of confidence. Placements were determined by applying the predefined decision criteria described in Table 3 to the evidence synthesized for each sensation and reported in Table 2; the matrix is a qualitative representation of the two confidence axes and does not constitute a numerical score or algorithmic scoring system. The matrix shows that high mechanistic confidence does not necessarily imply high translation confidence. Entries with HIGH mechanistic confidence, including TRPV1-mediated pungency, TRPM8-mediated cooling, astringency, and fatty mouthfeel, remain in the moderate-translation column. By contrast, sensations with moderate or debated mechanistic confidence, including kokumi, numbing, and oleogustus, remain difficult to predict mechanistically and are characterized by limited mechanism-based translation. This figure is a conceptual synthesis and does not represent experimental data.

### 9.1 Categorical confusion between taste, chemesthesis, and mouthfeel

The most pervasive challenge is categorical confusion between taste, chemesthesis, mouthfeel, and flavor (1, 25, 32). This is not merely terminological: it leads to inappropriate measurement choices, incomparable designs, and misleading interpretations (64, 77). Astringency is routinely described as a taste, when it is mechanistically a tactile–tribological phenomenon with no established role for taste-receptor cells (27, 28). Metallic sensation is classified as taste in some frameworks but has a well-evidenced retronasal olfactory component (12). Kokumi is treated as a taste quality in some publications and as a flavor modulator in others (32, 60). These inconsistencies can generate difficulties in comparing datasets, synthesizing evidence, and establishing consistent terminology for food research and product communication (1, 32, 60).

### 9.2 The oleogustus controversy

The proposal that FFA detection constitutes a sixth basic taste remains debated (29, 53). The available evidence–psychophysical clustering of FFA solutions (29), human oral-sensitivity data linked to CD36 variation (50), and cultured-human-cell CD36/GPR120 signaling (51)–supports perceptual distinctiveness and a plausible receptor contribution, but does not by itself establish oleogustus as an independent basic taste (53). The contribution of candidate receptors to human fatty-acid perception remains incompletely understood, and gustatory mechanisms need to be distinguished from olfactory, textural, and post-ingestive contributions to fat perception (53). Long-chain FFAs, including oleic and linoleic acids, have been reported to elicit an unpleasant taste when tested in isolation (29). We consider a possible spoilage-warning role to be a working hypothesis requiring direct testing. Unpleasantness alone does not establish this function, exclude nutrient sensing, or determine whether oleogustus qualifies as an independent basic taste (29, 53). Reported FFA detection thresholds should be discussed cautiously and not over-interpreted, since they vary widely with assay design, emulsification, and the difficulty of excluding olfactory cues (53); where they appear to exceed the free-fatty-acid levels of fresh food-grade fats, this further weakens the case for a routine nutrient-sensing role (53).

### 9.3 The absent-receptor problem for metallic sensation

Metallic sensation remains one of the least resolved mechanisms among the non-basic oral sensations discussed here (12, 55). No specific human metallic taste receptor has been identified (12, 55), the relative contributions of retronasal olfaction, gustation, and trigeminal chemesthesis have not been quantified across different metal-ion species (12, 55), and the oral-lipid-oxidation contribution has been supported primarily in model systems (12, 13), leaving formulation reliant on sensory panels rather than instrumentally guided design.

### 9.4 Ecological validity of receptor studies

The most structurally important methodological limitation is the ecological-validity gap between controlled laboratory conditions and real food consumption. Findings obtained under simplified experimental conditions may not fully reflect perception during eating (9, 15, 81). TRPV1, TRPA1, TRPM8, and CaSR are studied in heterologous cell-expression systems using pure agonists in physiological buffer (19, 21, 32, 49), an experimental context differing fundamentally from the oral cavity: a dynamic multi-phase system with continuous salivary flow, dilution and protein supplementation, masticatory shear, thermal gradients, mucoadhesive barriers, lipid and protein partitioning of bioactives, and microbial biotransformation (9, 15, 17). The effective concentration of any bioactive at the receptor surface *in vivo* depends on all of these simultaneously (9, 14), yet cell-based assays typically do not account for these interacting oral processes.

This is well illustrated by TRPM8 cooling, whose molecular mechanism is characterized in detail from receptor gating (47) to structural energetics (49). Biosensor responses agreed with human sensory findings in simplified solutions, but predictive validity across finished-food matrices was not assessed (81). Effective agonist concentration at the mucosa depends on matrix partitioning, salivary dilution, and mucoadhesion (9, 14, 17), so a high-potency compound in buffer may underperform a lower-potency agonist in a fat-containing matrix with superior mucosal bioavailability.

For astringency the problem takes a different form: it involves not taste-receptor activation but a tribological consequence of polyphenol–protein interactions (15, 26, 28). *In vitro* binding (ITC, fluorescence quenching) and precipitation (SDS-PAGE) assays operate on the correct molecular species but in the wrong physical context, lacking the salivary flow that renews the pellicle, the masticatory cycles that disrupt and replenish the film, and epithelial heterogeneity. Multiple salivary components, including mucins acting independently of PRPs, contribute in ways single-protein precipitation cannot capture (36), and reconstructed-epithelium tribology remains an *in vitro* approximation rather than direct human sensory evidence (37). A tribo-chemical framework makes the point explicit–chemical and physical processes are inseparable, so any single-dimension assay incompletely represents the mechanism (15)–and plant-derived biopolymers further modify lubrication independently of polyphenol content (17).

The problem is partly conceptual: mechanism is characterized at the molecular and subcellular level, while sensory experience is generated at the level of organ, organism, and cultural context (1, 3, 4). For some sensations, perception may be so context-dependent that no refinement of receptor assays will make them predictive on their own (9, 15, 81); progress will instead require systems-level and temporal sensory approaches that measure the integrated response directly (64, 66). Better *in vitro* models complement, rather than substitute for, dynamic human measurement (9, 15, 64), and until bridging studies become routine, formulation guidance will continue to rely on trained panels using temporally sensitive methods (64).

### 9.5 Methodological inadequacy for temporal sensations

Reliance on a single static QDA endpoint can be inadequate for sensations characterized by delayed onset, accumulation, adaptation, or persistence (64). Time-intensity and temporal-dominance methods provide complementary information about these temporal properties (64).

### 9.6 Individual variability and population representation

Inter-individual variability is large enough to affect study outcomes, yet trained panels exclude it by design, being optimized for product differentiation rather than population representation (70, 90). Individual responsiveness can itself shape acceptance: differences in astringency responsiveness are associated with differences in the acceptance of phenol-rich foods (90). Statistical approaches that model rather than average away such variation, including individual scaling and random-effects mixed models, are well established (91); a meta-analysis of 8,619 attributes from 369 sensory profiling datasets found significant scaling heterogeneity in 45% of attributes, indicating that individual differences in scale use are common rather than exceptional (91). In the literature examined here, however, trained-panel data are frequently reported as though they represented consumer response. This gap is most acute in the very product categories–plant-based foods, reduced-fat formulations, fortified beverages–where non-basic oral sensations are most commercially consequential (5, 7, 12).

## 10 Future perspectives and research directions

Progress will require genuine integration across food chemistry, oral biology, sensory science, neurophysiology, and computation. Improved *in vitro* mouth models incorporating salivary-secretion dynamics, thermal gradients, masticatory shear, multi-surface contact geometry, and realistic salivary-protein compositions would improve the ecological validity of mechanistic studies (8, 15, 17).

Personalized sensory prediction based on individual salivary proteomics and genetics is a medium-term but potentially transformative direction (92). Rapid, non-invasive quantification of an individual’s salivary protein composition (PRP isoform distribution, mucin glycoform profile, amylase activity) could allow prediction of susceptibility to polyphenol-induced astringency beyond population averages (35, 92).

Machine learning faces a fundamental limitation: training datasets are small, inconsistently measured, and generated using different sensory methods across laboratories (93). A coordinated effort to create standardized, openly accessible sensory reference datasets–including temporal, not only static, data combined with LC-HRMS and GC-MS composition data–would accelerate AI-based prediction (93).

Fundamental questions also remain open, including whether fat constitutes a sixth taste modality (94).

Connecting measurements already routine in food science to non-basic oral sensation is a related priority. Instrumental prediction of a sensory attribute is achievable within narrow, well-defined domains: shape characteristics have been used to model bitterness-related quality in pomelo (95). Processing interventions likewise produce measurable instrumental change, as combined marination and high-pressure processing at 300 MPa reduced hardness and chewiness in tuna steaks while sensory acceptability was retained (96), and raw-material composition varies systematically with genotype and agronomic practice, altering the phenolic, flavonoid and anthocyanin pools that constitute the chemical substrate for astringency (97). In none of these cases were non-basic oral sensations themselves measured, which illustrates the translational gap identified throughout this review: instrumental and compositional data are abundant, but are rarely linked to the specific oral sensations they are presumed to drive.

Standardized, validated reference compounds and lexical frameworks for each category, coordinated across academia and industry, remain a prerequisite for meaningful meta-analysis and are not yet achieved internationally (64, 77, 93). Controlled delivery and formulation strategies for pungency, cooling, and astringency require sensation-specific validation (10, 15, 81).

Finally, the three most contested mechanistic problems–oleogustus as an independent taste quality, kokumi mediated specifically by CaSR in human food contexts, and the receptor identity for metallic sensation–each have clearly defined experimental requirements (53, 55, 60). For oleogustus: human psychophysics under total nasal occlusion with pharmacological CD36/GPR120 antagonism in food-grade emulsions at physiologically realistic FFA concentrations (53). For kokumi: dose-response studies using CaSR-specific calcilytic antagonists before kokumi-active food stimuli in humans with confirmed peptide concentrations in the food source (57, 60, 61). For metallic sensation: systematic nasal-occlusion psychophysics across a metal-ion series with paired volatile profiling, extended to copper and zinc as well as iron (12, 13). These proposals are scientifically useful, but pharmacological receptor antagonism in human oral studies is not straightforward: ethical approval, food-grade suitability, safety, appropriate dosing, and technical feasibility would each have to be assessed case by case before such studies could proceed.

## 11 Conclusion

Non-basic oral sensations are important determinants of consumer acceptance, perceived product quality, and successful reformulation across several food sectors (5, 7, 14). Collectively, these sensations arise from diverse physiological and perceptual mechanisms, encompassing chemesthetic receptor activation, oral tribology, retronasal olfaction, and multimodal neural integration (1, 3, 15, 25). Their mechanistic diversity underscores that no single disciplinary perspective is currently sufficient to fully explain or predict their perception in complex food systems (8, 15, 53). Instead, meaningful advances will require an integrated framework that bridges molecular, physiological, sensory, and cognitive levels of analysis.

A central conclusion of this review is that the translational gap between *in vitro* receptor pharmacology and food sensory reality remains substantial and, on the evidence examined here, insufficiently addressed (9, 15, 81). Systematic syntheses exist within individual domains, including oral tribology and sensory perception (8) and the biological mediators of fat taste and smell (53), but the gap is visible directly in the two-axis assessment presented here: of the ten mechanistic entries in Table 2, four reached HIGH mechanistic confidence whereas none reached HIGH food-matrix translation confidence, and five were rated LOW (Table 2). EC_50_ values from TRP-channel assays, binding constants from buffer solutions, and precipitation indices from model systems provide mechanism-level insight but their predictive validity for sensation intensity or character across diverse real products remains incompletely established. This ecological-validity problem will not be resolved by more sophisticated cell assays alone; it requires designs that bridge across levels of biological organization, integrating oral tribology, dynamic mouth models, temporal sensory profiling, and individually calibrated prediction.

Several limitations bear on how these conclusions should be read. This review is integrative rather than systematic: literature was identified primarily through Google Scholar with backward reference-list searching, rather than by systematic searching of multiple bibliographic databases, and no screening log or formal inclusion count was maintained. The evidence base therefore reflects what this strategy identified, and completeness cannot be claimed. Emphasis was placed on literature published from 2015 onward, with older work retained selectively, including foundational primary discoveries, so earlier contributions outside those retained studies may be under-represented. The confidence ratings are qualitative judgments reached by author-level synthesis against the predefined criteria in Table 3; they are not a numerical scoring instrument, they were not assigned independently in duplicate, and no inter-rater agreement statistic is therefore reported. Citations supporting each rating are representative rather than exhaustive. Coverage across the nine sensations is also uneven, because the underlying literature is uneven: four of the ten mechanistic entries reached HIGH mechanistic confidence, whereas two remain DEBATED, and no entry reached HIGH food-matrix translation confidence, with five rated LOW. These constraints limit the strength of comparative claims between sensations, particularly for numbing, oleogustus, and kokumi, where human food-matrix evidence is sparsest. Building on the priorities identified in section “10 Future perspectives and research directions,” three needs are immediate: human sensory validation in real food matrices for the sensations currently rated LOW on translation confidence; standardized reference compounds and lexicons that permit comparison across laboratories; and openly accessible sensory datasets of sufficient size and temporal resolution to support predictive modeling.

The caution appropriate to a critical review is warranted: oleogustus, kokumi, and metallic sensation each present unresolved classification and mechanism problems that terminological convention cannot dissolve, TRPA1’s role in cold signaling remains debated and the fatty mouthfeel–oleogustus distinction must be maintained rather than collapsed for convenience. These caveats specify where investment in multimodal integration will yield the greatest return for food innovation and consumer health.
