# Supplementary material for: Beyond basic taste: mechanisms, measurement, and food-matrix translation of non-basic oral sensations
Source: Front Nutr. 2026 Oct 23;13:1962396. doi: 10.3389/fnut.2026.1962396 (PMC13740612; doi:10.3389/fnut.2026.1962396)
Supplement: Supplementary file 1 [file Table_1.docx]

# Supplementary Table 1. Evidence Table Underlying the Two-Axis Confidence Ratings

Each of the ten mechanistic entries assessed in Table 2 of the main text is set out below with its rating on both axes, the key human sensory evidence, the key mechanistic or model-system evidence, the principal limitations bearing on the rating, and representative references. Evidence types are labelled explicitly so that human psychophysical and trained-panel findings are not conflated with cultured-cell, animal, heterologous-expression, in vitro or computational evidence. Ratings follow the predefined criteria in Table 3 of the main text. Reference numbers correspond to the reference list of the main text.

| **Entry** | **Rating (mechanistic / translation)** | **Key human sensory evidence** | **Key mechanistic or model-system evidence** | **Principal limitations bearing on the rating** | **Representative references** |
| --- | --- | --- | --- | --- | --- |
| **Astringency** | **HIGH / MODERATE** | Trained-panel and instrumental study relating boundary friction and protein insolubility to panel astringency in acidic plant-protein-fortified smoothies (83); individual astringency responsiveness affects acceptance of phenol-rich foods (90); sensory responses to isolated pea, lupin and potato proteins with measurable haemodynamic neural responses (6). | Polyphenol–salivary-protein binding chemistry (27, 35); oral tribology of red wines (26); in vitro biotribometer on reconstructed oral-epithelial models expressing MUC1 isoforms (37); TRPV2 and TRPC5 as candidate targets in heterologous expression systems (43). | The proximal physicochemical step is well supported, but downstream neural transduction is unresolved and no dedicated astringency receptor is established. Evidence that tannins deplete oral mucins is contested (36). Single-compound binding assays predict matrix astringency poorly. | 6, 26, 27, 35, 36, 37, 43, 83, 90 |
| **Pungency (TRPV1)** | **HIGH / MODERATE** | Lingual capsaicin pre-treatment suppresses responses to tastants in humans (46); half-tongue two-alternative forced-choice testing identified chilli constituents that reduce perceived pungency (11); chemical–sensory comparison across food components (9); trained-panel evaluation of capsaicin-loaded nanocapsules (10). | TRPV1 identified as a capsaicin- and heat-activated channel (19); thermal activation threshold (24); luminescence-based human TRPV1 assay quantifying pungency in spicy foods (48). | Receptor identification is secure, but perceived pungency is strongly modified by matrix composition and endogenous suppressing constituents; assay–sensory correspondence has been shown only within limited food sets. | 9, 10, 11, 19, 24, 46, 48 |
| **Pungency (TRPA1)** | **MODERATE / LOW** | No dedicated human food-matrix psychophysical dataset was identified in this review. | Activation by pungent compounds and bradykinin (21); gating by covalent modification of reactive cytoplasmic cysteines (22); role in cold signalling reviewed and contested (47). | Non-electrophilic agonists may act through different routes; human food-matrix validation is sparse; the contribution to cold sensation remains debated. | 21, 22, 47 |
| **Cooling** | **HIGH / MODERATE** | Trained-panel ratings corresponded with the readout of a human TRPM8 cell-based biosensor for menthol, WS-3 and WS-23 and their binary mixtures, tested in simplified solutions (81). | TRPM8 cloned from rat trigeminal neurons and characterised as a cold- and menthol-activated channel in heterologous expression (20); thermal-threshold mechanism (24); review of TRPM8 in innocuous cooling and of the debated role of TRPA1 in cold (47); human TRPM8 cell-based biosensor with Chou–Talalay analysis of menthol, WS-3 and WS-23 interactions (81); structural energetics of cold sensitivity (49). | Biosensor responses agreed with human sensory findings in simplified solutions, but predictive validity across finished-food matrices was not assessed (81). | 20, 24, 47, 49, 81 |
| **Metallic sensation** | **MODERATE / MODERATE** | Human psychophysics with nasal occlusion: FeSO₄ metallic ratings fell to baseline in two experiments; comparable reductions were not observed for CuSO₄ or ZnSO₄ (12). | Identification of metallic-smelling 1-octen-3-one and 1-nonen-3-one from ferrous sulfate solutions (13); review of metal-salt flavour profiles (55). | Perception is multimodal and heterogeneous across metal salts; no dedicated human metallic receptor has been identified and no validated instrumental proxy exists. | 12, 13, 55 |
| **Fatty mouthfeel** | **HIGH / MODERATE** | Systematic review of studies relating oral tribology to sensory perception (8); trained-panel sensory work on food gels differing in fat-droplet characteristics (7). | Fat-droplet size, interfacial composition and shear stability govern friction at the tongue–palate interface (7); oral lubrication performance of plant-derived polymers (17); mouthfeel physiology and biochemistry (14). | The specific mechanoreceptor population is unresolved; tribology alone does not predict the full multidimensional fat percept. Distinct from oleogustus: CD36/GPR120 are not required for the lubrication-based component. | 7, 8, 14, 17 |
| **Oleogustus** | **DEBATED / LOW** | Human psychophysics supporting the perceptual distinctiveness of free fatty acids (29); in 21 obese participants a CD36 variant (rs1761667) associated with lower expression corresponded to about eight-fold higher oral detection thresholds (50); GPR120 agonists enhanced fatty orosensation when added to a fat-containing system but did not evoke it alone (54). | Cultured human taste-bud cells show CD36- and GPR120-mediated signalling, with GPR40 undetectable in human and rat taste-bud cells (51); CD36-dependent gut release of secretin and cholecystokinin indicates a post-ingestive role (52); systematic review of biological mediators (53). | Sensory quality at detectable concentrations is predominantly rancid and aversive; thresholds may exceed those of fresh food-grade fats; retronasal aroma from oxidation products is difficult to exclude; basic-taste status remains contested (94). | 29, 50, 51, 52, 53, 54, 94 |
| **Tingling** | **MODERATE / LOW** | Psychophysical evaluation of a sanshool derivative in humans characterising the tingle percept (31). | Hydroxy-α-sanshool excites sensory neurons by inhibiting KCNK3, KCNK9 and KCNK18 two-pore potassium channels (30; animal and heterologous systems). | Mechanistic characterisation is largely model-system based; translation to whole-food matrices is poorly characterised; any TRPV1/TRPA1 contribution at high concentrations is minor and uncertain. | 30, 31 |
| **Numbing** | **DEBATED / LOW** | Sensory characterisation of pomelo peel numbing attributing the percept to coumarins rather than alkylamides (56). | Associated with sanshool-related KCNK modulation (30, 31); no numbing-specific transduction pathway demonstrated. | No numbing-specific mechanism is established, and food-derived numbing arises from chemically distinct compound classes, indicating mechanistic heterogeneity. | 30, 31, 56 |
| **Kokumi** | **MODERATE / LOW** | γ-Glutamyl-valyl-glycine increased umami, mouthfulness and mouth-coating in chicken consommé in descriptive analysis (59); γ-glutamyl peptides elicit kokumi in human taste testing, and the CaSR antagonist NPS-2143 suppressed the kokumi intensity of glutathione and γ-glutamyl-valyl-glycine in model solutions (32); early characterisation of continuity, mouthfulness and thickness (23). | CaSR-expressing taste cells respond to kokumi substances (57; mouse taste cells); cryo-EM structure of CaSR with γ-glutamyl-valyl-glycine (61); enzymatic generation of γ-glutamyl peptides validated by taste-receptor assay (62); glutathione-derived Maillard reaction products altered CaSR activation in vitro in a sugar- and temperature-dependent manner (63); classification as taste modulators (60). | CaSR involvement is supported but exclusivity and sufficiency for human kokumi perception are unproven; several newer peptides are supported only by in vitro or computational evidence; predictive value in complex foods is not established. The antagonist experiment (32) used model solutions and does not establish predictive validity across finished-food matrices. | 23, 32, 57, 59, 60, 61, 62, 63 |

**Evidence-type note.** Human sensory evidence comprises human psychophysics and trained-panel food sensory studies. Mechanistic or model-system evidence comprises heterologous receptor expression, cultured human taste-bud cells, animal models, ex vivo and reconstructed-epithelium preparations, in vitro tribology, and computational studies. No cell, animal, in vitro or computational result is treated as direct evidence of human perception. Ratings are qualitative author-assigned judgements applying the predefined criteria in Table 3, not a numerical score.
